# Basophils activate oncostatin M receptor–expressing vagal sensory neurons

**DOI:** 10.1016/j.jaci.2025.10.027

**Published:** 2025-11-07

**Authors:** Jo-Chiao Wang, Kicheon Park, Anais Roger, Amin Reza Nikpoor, Theo Crosson, Hoon James Sunwoo, Eva Kaufmann, Moutih Rafei, Eric H. Chang, Sebastien Talbot

**Affiliations:** a Department of Pharmacology and Physiology, Université de Montréal, Montreal, Quebec; b Institute for Bioelectronic Medicine, Feinstein Institutes for Medical Research, Northwell Health, Manhasset; c Department of Biomedical and Molecular Sciences, Queen’s University, Kingston, Ontario; d Department of Physiology and Pharmacology, Karolinska Institutet, Stockholm; e Meakins-Christie Laboratories, RI-MUHC, and Faculty of Dental Medicine and Oral Health Sciences, McGill University, Montreal, Quebec.

**Keywords:** Neuroimmunology, asthma, allergy, nociceptor neurons, OSM, basophils

## Abstract

**Background:**

Vagal sensory neurons (VSNs) relay signals from internal organs to the brainstem via the vagus nerve. Published transcriptomic data sets suggest that VSNs express Mas-related G protein–coupled receptor family member D and oncostatin M (OSM) receptor, markers also found in dorsal root ganglia–resident pruriceptor neurons. However, the cellular source of OSM and its potential interactions with VSNs remain unknown.

**Objective:**

We sought to validate the expression of pruritogen receptors on VSNs, identify basophils as a source of OSM, and determine how OSM modulates VSN activity.

**Methods:**

Pruritogen receptor expression on VSNs was validated by immunofluorescence staining, and VSN responses to pruritogens were assessed using *in vitro* and *in vivo* calcium imaging. Mouse models of allergic airway inflammation, fluorescence-activated cell sorting, quantitative PCR, and ELISA were used to evaluate OSM expression and production by lung basophils.

**Results:**

Immunofluorescence and quantitative PCR confirmed that airway-innervating VSNs express OSM receptor and that lung basophils are enriched for *Osm* transcripts. Lung-resident basophils produced OSM following FcεRIα engagement, with production further enhanced after sensitization to house dust mite (*Dermatophagoides pteronyssinus*) or the fungal allergen *Alternaria alternata*. *In vitro* and *in vivo* calcium imaging revealed that OSM sensitizes multiple populations of VSNs.

**Conclusions:**

These findings identify a novel mechanism of communication between basophils and VSNs during type I hypersensitivity reactions, such as those occurring in allergic asthma.

Airway sensory innervation is primarily projected from the jugular and nodose ganglia along the vagus nerve.^1–3^ In mice, these 2 ganglia are anatomically fused and collectively referred to as the jugular-nodose complex (JNC).^4^ Jugular neurons share both developmental and molecular features with dorsal root ganglia (DRG)-resident somatosensory neurons, including a neural crest origin and expression of the transcription factor PR domain zinc finger protein 12 (*Prdm12*).^4^ In contrast, nodose neurons derive from the neural placode, express the transcription factor paired-like homeobox 2B (*Phox2b*), and predominantly mediate interoceptive functions.^5^ Over the past few decades, research on vagal sensory neurons (VSNs) has largely focused on those expressing transient receptor potential vanilloid 1 (TRPV1), because of their resemblance to pain-mediating DRG nociceptors (nociceptor neurons).^6,7^ However, recent single-cell transcriptomic analyses indicate that certain jugular neurons exhibit transcriptional profiles similar to nonpeptidergic neurons—those involved in itch sensation and thus also known as pruriceptor neurons.^8^

Pruriceptor neurons are DRG-resident primary afferent cells that detect itch-inducing agents (pruritogens) and relay itch signals through the spinal cord to the somatosensory cortex. Single-cell RNA sequencing (scRNA-seq) has categorized these pruriceptor neurons into 3 main types: (1) NP1, which expresses Mas-related G protein–coupled receptor D (*Mrgprd*) and lysophosphatidic acid receptor 3 (*Lpar3*), mediating nonhistaminergic itch; (2) NP2, which expresses *Mrgpra3* and the GDNF family receptor α subunit 1; and (3) NP3, which expresses IL-31 receptor α (*Il31ra*) and oncostatin M receptor β (*Osmr*), as well as somatostatin (*Sst*) and natriuretic peptide precursor B (encoded by *Nppb*).^9^ Both NP2 and NP3 populations express OSMR and contribute to histaminergic itch.^9^ Notably, scRNA-seq of VSNs has revealed jugular neuron clusters that closely resemble these pruriceptor subsets.^8^ Although cytokine-mediated itch generation and pruriceptor sensitization—often driven by IL-33, thymic stromal lymphopoietin, and oncostatin M (OSM)^10,11^—have been well studied in skin conditions such as atopic dermatitis and psoriasis, relatively little research has addressed similar mechanisms in VSNs expressing these receptors.

Basophils and mast cells have long been recognized as key effector cells in allergic reactions, including itch.^12–14^ Despite differences in their maturation and tissue localization, both cell types share several functional traits. One hallmark is activation on antigen-induced crosslinking of IgE bound to the high-affinity receptor FcεRIα on their membranes, which leads to the release of mediators such as histamine, serotonin, cysteinyl leukotrienes, and prostaglandins.^15^ Basophils, in particular, are noted for their robust expression and rapid release of T_H_2 cytokines, including IL-4 and IL-13.^15^ In severe asthma, basophils can also be activated by bacterial products such as *Staphylococcus aureus* enterotoxins,^16^ and they may show increased expression of receptors for alarmins (IL-25, IL-33, and thymic stromal lymphopoietin).^17^ Recent scRNA-seq studies further demonstrate that mouse lung basophils express elevated levels of IL-6 family cytokines, including *Il6*, *Lif*, and, interestingly, *Osm* (the cognate ligand for OSMR).^18–20^

Clinically, basophil numbers correlate with a history of asthma,^21^ nighttime cough, and cold air–induced wheezing and coughing,^22^ suggesting that basophils may contribute to neurogenic symptoms. Mechanistically, basophils facilitate acute itch via the synthesis and secretion of leukotriene C4 and IL-4,^12,23^ which directly activate pruriceptor neurons, forming a basophil-neuron axis in the skin. Whether a comparable axis exists in the airways and influences the pathophysiology of allergic lung diseases remains an open question.

Given that OSM has been implicated in thermal hypersensitivity and chronic itch,^11,24^ and that OSMR is expressed on MrgprA3^+^ NP2 and *Nppb*^+^ NP3 pruriceptor neurons—as well as on subsets of VSNs—we hypothesized that OSM may facilitate crosstalk between basophils and VSNs during allergic airway inflammation.^25^ Here, we investigate the identity of pruritogen receptor–expressing VSNs and evaluate whether OSM can modulate their activity.

## METHODS

### Animals and experimental procedures

All animal experiments were conducted in accordance with protocols approved by the Institutional Animal Care and Use Committee of Université de Montréal, the Queen’s University Animal Care Committee, and the Feinstein Institutes for Medical Research. All procedures complied with the guidelines of the Canadian Council on Animal Care and the National Institutes of Health Guide for the Care and Use of Laboratory Animals. Mice were housed in individually ventilated cages under a 12-hour light/dark cycle, with *ad libitum* access to food and water.

Mouse strains included C57BL/6J (000664), *Phox2b*^*cre*^ (016223), *Tac1*^*cre*^ (021877), *Scn10a*^*cre*^ (036564), *Slc17a6*^*cre*^ (028863), *GCaMP6f*^*fl/fl*^ (028865), *tdTomato*^*fl/fl*^ (007914), and *Salsa6f*^*fl/fl*^ (031968), all originally obtained from The Jackson Laboratory (Bar Harbor, Me) and bred in-house. For calcium imaging experiments, lineage-specific reporter mice were generated by crossing the respective Cre-driver lines with *GCaMP6*^*fl/fl*^, *tdTomato*^*fl/fl*^, or *Salsa6f*^*fl/fl*^ reporter lines. Male and female mice aged 6 to 10 weeks were used for all experiments unless otherwise specified.

### Ovalbumin-induced lung inflammation

For the ovalbumin (OVA)-induced allergic airway inflammation model, C57BL6 mice were sensitized by intraperitoneally injecting an emulsion of grade V OVA (A5503; 100 μg/dose; Sigma-Aldrich, St Louis, Mo) and Imject Alum (77161; 1 mg/dose; Thermo Fisher Scientific, Waltham, Mass) on days 0 and 7, followed by intranasal challenges with OVA (50 μg/dose) with or without fine particulate matter (NIST SRM 2786; 20 μg/dose; NIST, Gaithersburg, Md) from day 14 to 16. Control mice were sensitized but not challenged. The mice were sacrificed on day 17 to harvest tissues.

### House dust mite–induced lung inflammation

C57BL6 mice received daily intranasal injections of house dust mite (HDM) extract (02.01.85; 20 μg/dose; CiteQ Biologics) from day 0 to 5 as sensitization and from day 8 to 10 as challenges. PBS was injected into control mice. Mice were sacrificed on day 11 following anesthesia with intraperitoneally injected urethane (94300; 2 g/kg body weight; Sigma-Aldrich) to harvest tissues.

### *Alternaria alternata*–induced lung inflammation

C57BL6 mice received daily intranasal injections of *A alternata* media (09.01.26; 100 μg/dose; CiteQ Biologics, Groningen, Netherlands) from day 0 to 5 as sensitization and from day 8 to 10 as challenges. PBS was injected into control mice. Mice were sacrificed on day 11 following anesthesia with intraperitoneally injected urethane (94300; 2 g/kg body weight; Sigma-Aldrich) to harvest tissues.

### *In silico* analysis of gene expression in VSNs

Gene expression data were extracted from the Online Repository materials of the study by Kupari et al^8^ and plotted using GraphPad Prism, with cluster definitions taken from the original publication. Data from GSE192987^26^ were downloaded from the National Center for Biotechnology Information Gene Expression Omnibus and reanalyzed using the Seurat package in R. Quality control filtering retained cells with more than 200 and fewer than 8000 detected genes, and with less than 5% of genes being mitochondrial. After quality control, 63,945 cells remained and were grouped into 30 clusters at a resolution of 0.5 using Uniform Manifold Approximation and Projection (UMAP), with cells expressing the indicated genes (transcripts per million [TPM] > 0) color-labeled on the UMAP. For the GSE278510 data set, quality control criteria matched those used in the original study,^27^ yielding 16,931 cells grouped into 19 clusters at a resolution of 0.45 (UMAP). Data from GSE223355 were obtained from the Online Repository information of the original article,^27^ and FPKM (fragments per kilobase of transcripts per million map reads) values for *Trpv1* and *Osmr* were plotted.

### Neuron culture

JNC and DRG were extracted and dissociated as previously described.^28,29^ Briefly, JNC was collected following exsanguination, whereas DRG was collected after the decapitation of anesthetized mice. Ganglia were placed into a digestion buffer containing 1 mg/mL (325 U/mL) collagenase type 4 (LS004189; Worthington, Lakewood, NJ), 2 mg/mL (1.8 U/mL) Dispase II (04942078001; Sigma-Aldrich), and 250 μg/mL (735.25 U/mL) DNase I (11284932001; Sigma-Aldrich) (see Table E1 in this article’s Online Repository at www.jacionline.org), prepared in supplemented Dulbecco modified Eagle medium (DMEM) (see Table E2 in this article’s Online Repository at www.jacionline.org) and was incubated at 37°C for 60 minutes. Mechanical dissociation was performed by pipetting the digested tissue with pipette tips of decreasing diameter and finishing with 25-gage needles, followed by density gradient centrifugation with 150 mg/mL BSA (SH30574.02; Hyclone; PBS solution) over a PBS layer. Cells were seeded onto glass-bottom dishes (81218; Ibidi) precoated with 50 μg/mL laminin (L2020; Sigma-Aldrich) and 100 μg/mL poly-d-lysine (P6407; Sigma-Aldrich) (see Table E3 in this article’s Online Repository at www.jacionline.org) and cultured overnight in Neurobasal-A media supplemented with 50 ng/mL NGF (nerve growth factor; 13257–019; Thermo Fisher Scientific) and 2 ng/mL GDNF (glial cell line-derived neurotrophic factor; NBP2–61336; Novus Biologicals, Centennial, Colo) (see Table E4 in this article’s Online Repository at www.jacionline.org) before recording calcium imaging.

### Isolating airway-innervating VSNs

Isolation of airway-innervating Na_V_1.8^+^ VSNs was conducted as previously described.^27^ Briefly, *Scn10a*^*tdTomato*^ mice received an intranasal injection of DiD tracer (D7757; 200 μM in 50 μL; Invitrogen, Carlsbad, Calif) on day 0 to label airway-innervating neurons. Fourteen days later, JNCs were collected and dissociated as described earlier. Airway-innervating (DiD^+^) and non–airway-innervating (DiD^−^) Na_V_1.8^+^ neurons (tdTomato^+^) were then fluorescence-activated cell sorting (FACS)-purified, followed by RNA extraction and quantitative PCR (qPCR) to assess transcript levels of the indicated genes.

### Bronchoalveolar lavage fluid and lung tissue harvest

Bronchoalveolar lavage was performed on mice anesthetized as previously described,^27^ with incisions made to the trachea. The mice were lavaged twice with 1 mL of PBS or FACS buffer (2% FBS and 1 mM EDTA in PBS) using a Surflo ETFE IV Catheter 20G × 1ʺ (SR-OX2025CA; Terumo Medical Products). The collected lavage fluid was centrifuged at 350*g* for 6.5 minutes. The supernatant was collected for ELISA, and the cell pellets were resuspended, subjected to RBC lysis (TNB-4300-L100 [Cytek, Fremont, Calif] or A1049201 [Gibco, Thermo Fisher Scientific]), and stained for surface markers for flow-cytometric analysis. Lungs were harvested following a diaphragm incision and transcardial perfusion with 10 mL of PBS, minced with razor blades, and collected into TRIzol Reagent (15596026; Invitrogen) for RNA extraction or into a digestion buffer (see Table E5 in this article’s Online Repository at www.jacionline.org; 1.6 mg/mL collagenase type 4 and 100 μg/mL DNase I in supplemented DMEM [as in Table E2]) to prepare a single-cell suspension. This suspension was obtained through 45 minutes of enzymatic digestion at 37°C, with mechanical dissociation using 18-gage needles performed midway through incubation (30 minutes), followed by filtering through a 70-μm nylon mesh, and RBC lysis. For flow-cytometric analysis or FACS, cells were resuspended in FACS buffer; for *in vitro* stimulation, cells were resuspended in FBS-supplemented DMEM, seeded into 96-well plates, and cultured at 37°C with 5% CO_2_ for the specified time before supernatant collection.

### *In vitro* stimulation of lung cells

Dissociated lung cells were cultured in 96-well tissue culture plates treated to support cell growth, with a density of 1 × 10^6^ cells/well. The cells were treated with 1 μg/mL MAR-1 (134304; BioLegend, San Diego, Calif), 1 μg/mL Ba13 (142215; BioLegend), or a mixture of corresponding isotype antibodies (see Table E6 in this article’s Online Repository at www.jacionline.org). Supernatants were collected at 3 hours and 24 hours poststimulation in the initial experiment. The 3-hour time point was chosen to compare cytokine release from cells treated under different conditions, because the basal release of cytokines is lower at this time point.

### Fluorescence-activated cell sorting

Single-cell suspensions derived from bronchoalveolar lavage fluid (BALF), or lung samples, were stained with Ghost Dye Violet 510 (13–0870-T100; Cytek, Fremont, Calif) and antibody cocktails in PBS at 4°C for 30 minutes, followed by fixation with 10% neutral buffered formalin (HT501128; Sigma-Aldrich) at room temperature for 15 minutes before flow-cytometric data acquisition. For assessing eosinophil and neutrophil infiltration, BALF cells were stained with fluorochrome-conjugated antibodies targeting CD45 (30-F11), CD90.2 (53–2.1), CD11b (M1/70), CD11c (N418), Ly6C (HK1.4), Ly6G (1A8), and Siglec-F (1RNM44N). For basophil sorting, cells from BALF or lung were stained with antibodies against CD45 (30-F11), CD90.2 (53–2.1), CD49b (DX5), FcεRIα (MAR-1), c-kit (2B8), Siglec-F (1RNM44N), and lineage markers including CD3ε (145–2C11), CD19 (1D3/CD19), NK1.1 (PK136), and CD11c (N418). These antibodies were sourced from BioLegend or Thermo Fisher Scientific and diluted (see Table E6). For FACS, cells were resuspended in FACS buffer poststaining without fixation, filtered again using Falcon round-bottom tubes with a cell strainer cap (35-μm strainer; 38030; Corning), and analyzed. Data acquisition was performed using a FACS Canto II (BD Biosciences, Franklin Lakes, NJ,) or CytoFLEX S (Beckman Coulter, Brea, Calif) flow cytometer. Cell sorting was conducted on BD FACSAria III or BD FACSAria Fusion. Analyzer and sorter configurations are provided in Tables E7–E10 (in the Online Repository available at www.jacionline.org).

### Immunofluorescence staining

The JNCs were dissected, fixed in 4% paraformaldehyde for 1 hour at room temperature, and subsequently washed with 1× PBS. The tissues were then cryoprotected by overnight incubation in 30% sucrose at 4°C and stored at −20°C in OCT (optimal cutting temperature) compound (14–373-65; Fisherbrand, Waltham, Mass) until use. Frozen JNC sections (12 -μm thick) were cut using a cryostat and collected on Superfrost Plus slides (12–550-15; Fisherbrand). For immunolabeling, tissue sections were saturated and permeabilized for 1 hour at room temperature with a solution containing 3% BSA, 0.3% Triton X-100, and 10% donkey serum in 1× PBS. Next, the following primary antibodies were applied: rabbit anti-OSMR (1:300; EPR28222–64; Abcam, Cambridge, UK) and anti–β3-tubulin (1:300; 657408; BioLegend). After overnight incubation at 4°C, sections were washed several times with 1× PBS, and secondary antibodies (anti-rabbit Alexa Fluor 488, 1:500; 711–545-152; Jackson ImmunoResearch) were added for 1 hour at room temperature. Finally, the sections were mounted in ProLong Gold Antifade Mountant with 4'−6-diamidino-2-phenylindole (P36931; Thermo Fisher Scientific). Images were acquired using a Nikon AX/AX R confocal microscope (Nikon, Tokyo, Japan) and analyzed in Fiji for ImageJ (version 1.53f51) with the cell counter plugin.

For staining with retrograde labeling, C57BL/6 mice were intranasally injected with 50 μL of 0.4 mM Fast Blue (17740; Polysciences, Warrington, Pa) diluted in 1% dimethyl sulfoxide in 1× PBS. Four days later, JNCs were harvested and fixed in 4% paraformaldehyde for 1.5 hours at 4°C. After washing with 1× PBS, samples were cryoprotected overnight at 4°C in 30% sucrose, embedded in OCT compound (14–373-65; Fisherbrand), and stored at −20°C until use. Frozen ganglia were sectioned (12 μm) onto Superfrost Plus slides (12–550-15; Fisherbrand). For immunolabeling, tissues were blocked and permeabilized for 1 hour at room temperature in 1× PBS containing 3% BSA, 0.2% Triton X-100, and 10% donkey serum. Primary antibodies—rabbit anti-OSMR (1:300; EPR28222–64; Abcam) and goat anti-Phox2b (1:200; AF4940-SP; Novus Biologicals)—were applied and incubated overnight at 4°C. After washing, secondary antibodies—anti-rabbit Alexa Fluor 594 and anti-goat Alexa Fluor 488 (1:500; Jackson ImmunoResearch, West Grove, Pa)—were added for 1 hour at room temperature. Sections were then incubated for 1 hour with isolectin GS–isolectin B4 conjugated to Alexa Fluor 674 (1:300; I32450; Thermo Fisher Scientific). Finally, slides were mounted in ProLong Gold Antifade Mountant (P36930; Thermo Fisher Scientific) and images were acquired as described earlier.

### *In vivo* vagal ganglion calcium imaging

Vagal ganglion calcium imaging was performed as described previously.^30,31^ Briefly, 8- to 12-week-old male and female *Slc17a6*^*GCaMP6f*^ (*Slc17a6*^*cre*^::*GCaMP6f*^*fl/wt*^) mice were anesthetized with isoflurane (1.75% in 1 L/min O_2_ flow) and placed in a supine position on a small animal physiological monitoring system equipped with integrated temperature control (Harvard Apparatus, Holliston, Mass). The cervical region was shaved and sterilized with 70% ethanol. An incision was made in the submandibular area, and the vagal ganglion was exposed under a branch of the carotid artery after separating and retracting the masseter muscles. A custom retractor made from a 40 -μm EZ-Flow cell strainer (Foxx Life Sciences, Salem, NH) was used to support the vagal ganglion, which was continuously bathed in saline. A Miniscope V4^32^ with a coverslip-mounted baseplate was then lowered onto the ganglion using a stereotaxic frame. Before image acquisition, Miniscope DAQ settings were optimized to the following parameters: 70 LED exposure, 3.5 gain, 20 FPS, 0.80 alpha, and 0.10 to 0.15 beta.

For calcium imaging analysis, raw fluorescence data were processed using the open-source Python-based pipeline Calcium Imaging Analysis (CaImAn).^33^ First, a nonrigid motion correction algorithm (NoRMCorre) was applied, followed by the constrained nonnegative matrix factorization for microendoscope data (CNMF_E) for region-of-interest detection. The resulting CaImAn output was further processed using a custom Python package that computed the integral of each detected calcium transient. To assess the effect of OSM on lung-innervating VSNs, recombinant mouse OSM (100 ng/mL) was nebulized intranasally at a flow rate of 0.25 mL/min and an airflow of 6.7 L/min for 5 minutes. The MrgprA3 agonist chloroquine (1 mM) was applied to the vagus nerve before or after OSM administration.

### *In vitro* calcium imaging

Cultured neurons from C57BL6, *Phox2b*^*Salsa6f*^ (*Phox2b*^*cre*^::*Salsa6f*^*fl/wt*^), or *Tac1*^*Salsa6f*^ (*Tac1*^*cre*^::*Salsa6f*^*fl/wt*^) were loaded with the calcium indicator dye, 5 μM Fura-2 AM (acetoxymethyl) (34993; Cayman Chemical Company, Ann Arbor, Mich), and incubated at 37°C for 40 minutes. After incubation, they were washed 4 times with standard external solution (SES; C-3030F; Boston BioProducts, Braintree, Mass) and subsequently imaged in SES. Neurons from *Scn10a*^*GCaMP6f*^ (*Scn10a*^*Cre*^::*GCaMP6fl/wt*) mice were washed with SES and immediately used for imaging. In experiments using Salsa6f reporter lines, the fluorescent signal from GCaMP6f completely coincided with tdTomato^+^ cells (data not shown). The data recorded with signals from Fura-2 were used for further analysis. Agonists were diluted in SES (see Table E11 in this article’s Online Repository at www.jacionline.org) and administered through a ValveLink8.2 system (Automate Scientific, Berkeley, Calif) with 250-μm Perfusion Pencil tips (Automate Scientific), facilitated by Macro Recorder (Bartels Media, Berlin, Germany). SES was continuously flowed during intervals between drug injections to wash out the drugs. Imaging for Fura-2 experiments was conducted using an S Plan Fluor ELWD 20X objective lens (Nikon, Tokyo, Japan) to enhance UV light passage, whereas GCaMP6f experiments used an S Plan Fluor LWD 20X lens (Nikon) for improved resolution. Images were captured at 3- or 4-second intervals using pco.edge 4.2 LT (Excelitas Technologies, Pittsburgh, Pa), Prime 95B (Teledyne Photometrics, Tucson, Ariz), or Orca Flash 4.0 v2 (Hamamatsu Photonics, Shizuoka, Japan) sCMOS cameras. Imaging was performed on ECLIPSE Ti2 inverted microscopes (Nikon). Regions of interest were manually delineated on NIS-Elements software (Nikon), and the F340/F380 ratio or GFP (green fluorescent protein) measurements were exported to Excel (Microsoft, Redmond, Wash) for further analysis. Data were condensed into a maximum value every 15 seconds for all analyses.^27,34^

### Real-time qPCR

Sorted cells, freshly minced lung tissues, or digested lung single-cell suspensions were lysed using TRIzol Reagent (15596018; Thermo Fisher Scientific)^35,36^ and stored at −80°C before RNA extraction. RNA from sorted cells was extracted using PureLink RNA Micro Scale kits (12183016; Thermo Fisher Scientific), whereas RNA from lung tissues or lung cell suspensions was extracted using E.Z.N.A. Total RNA Kit I (R6834; Omega Bio-tek, Norcross, Ga). All RNA extractions were conducted according to the manufacturer’s instructions, following phenol-chloroform phase-based purification and mixing with equal volumes of isopropanol. cDNA synthesis was carried out using SuperScript VILO Master Mix (11755050; Invitrogen, Carlsbad, Calif) with 1 to 2 μg of RNA template for each reaction. qPCR was performed using PowerUp SYBR Green Master Mix (A25742; Applied Biosystems, Foster City, Calif), 50 to 100 ng of cDNA templates, and 200 nM of respective primers (see Table E12 in this article’s Online Repository at www.jacionline.org) on a Mic qPCR Cycler (Bio Molecular Systems) or CFX Opus Real-Time PCR System (Bio-Rad Laboratories, Hercules, Calif).

### ELISA

Cytokine levels in supernatant collected from BALF or cell culture were determined with commercially available ELISA kits from BioLegend and R&D Systems (Minneapolis, Minn) (see Table E13 in this article’s Online Repository at www.jacionline.org).

### Data availability

Information and raw data are available from the lead contact on reasonable request.

### Statistical analysis

*P* values less than or equal to .05 were considered statistically significant. One-way ANOVA, 2-way ANOVA, and Student *t* tests were performed using Prism (GraphPad, San Diego, Calif). Seurat packages on R were used for analyzing scRNA-seq data sets using RStudio.

### Replicates

Replicates (n) are described in the figure legends and represent the number of animals for *in vivo* data. For *in vitro* data, replicates can be culture wells or dishes, animals, fields of view (microscopy), or neurons (calcium microscopy), but will always include different preparations from different animals to ensure biological reproducibility.

## RESULTS

**FIG 1.**
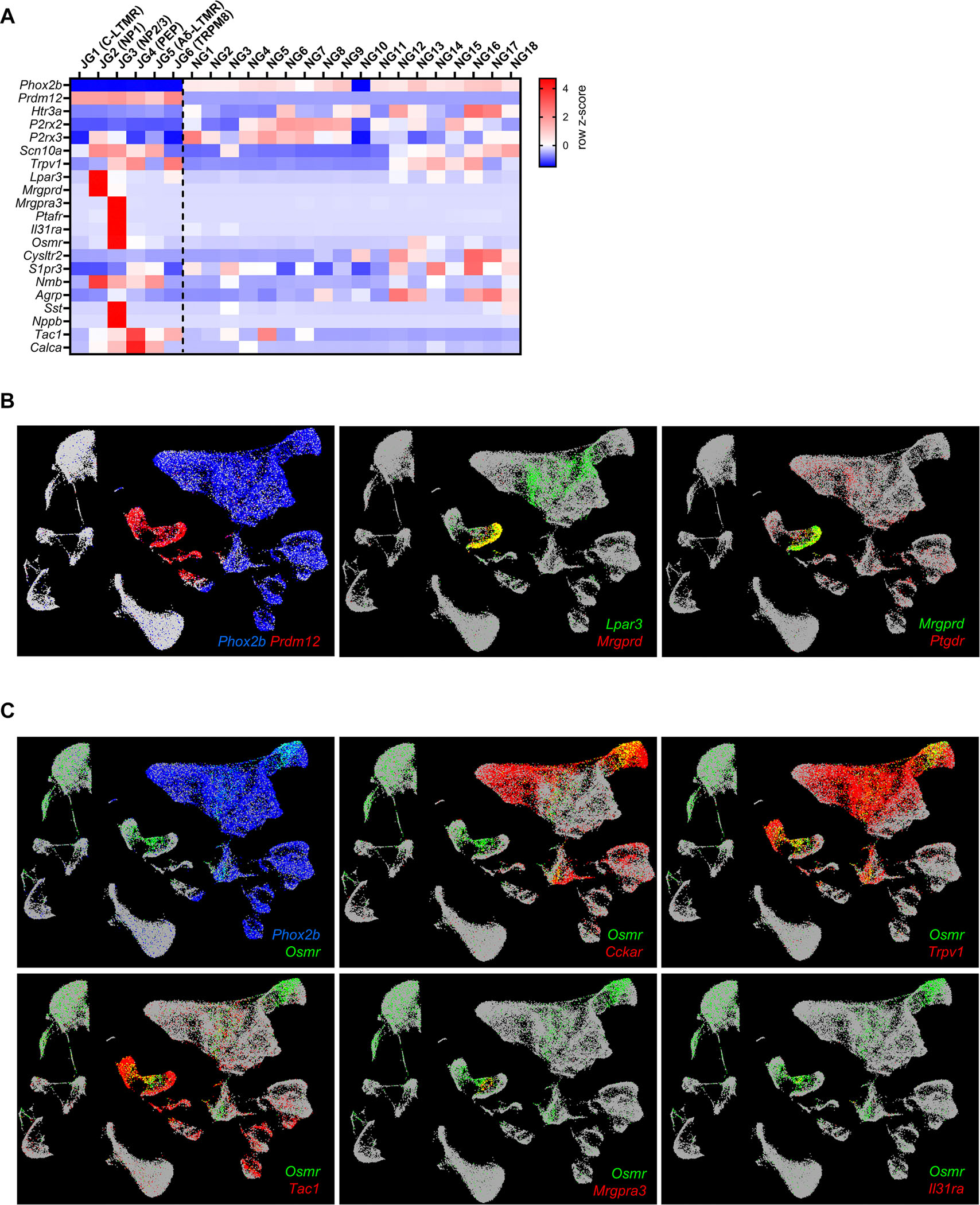
scRNA-seq data set revealing *Mrgprd* and *Osmr*-expressing VSNs. **A,** *In silico* analysis of the data set GSE124312 generated a heatmap that shows the transcript expression levels of lineage-defining transcription factors (*Phox2b* and *Prdm12*), ion channels *(Htr3a*, *P2rx2*, *P2rx3*, *Scn10a*, and *Trpv1*), pruriceptor markers (*Lpar3*, *Mrgprd*, *Mrgpra3*, *Il31ra*, *Osmr*, *Cysltr2*, and *S1pr3*), and neuropeptides (*Nmb*, *Agrp*, *Sst*, *Nppb*, *Tac1*, and *Calca*). **B** and **C,** UMAP plots generated from the database GSE192987 feature pseudocolor highlighting of cells expressing specific genes, illustrating Phox2b-expressing placodal neurons and Prdm12-expressing neural crest neurons. Neurons labeled as NP1, which express *Lpar3* and *Mrgprd*, are shown in plots (Fig 1, B). Meanwhile, cells expressing *Osmr*, which coexpress *Cckar*, *Trpv1*, *Tac1*, *Mrgpra3*, and *Il31ra*, are depicted in the plot (Fig 1, C). In Fig 1, A, the data are presented as a heatmap displaying each cluster’s *z* scores of average gene expressions. Experimental details and cell clustering are defined in Kupari et al.^8^ In Fig, 1, B and C, the data are shown as colored dots, and the cells are labeled with nonzero values in counts per 10,000. The UMAP is derived from the database GSE192987, with experimental details outlined in Zhao et al.^26^

scRNA-seq data from Kupari et al (GSE124312^8^) indicate that NP1 pruriceptor markers *Lpar3* and *Mrgprd* are enriched in the jugular ganglion cluster JG2, whereas NP2 and NP3 markers—including *Il31ra*, *Mrgpra3*, *Osmr*, *Sst*, and *Nppb*—are found in cluster JG3 (Fig 1, A). NP2- and NP3-like jugular neurons appear as a single cluster, likely because of their shared gene expression profiles and low cell numbers (Fig 1, A). To determine whether these markers colocalize within individual neurons, we performed an *in silico* analysis of Zhao’s data set (GSE192987^26^). This analysis revealed NP1-like VSNs coexpressing *Lpar3* and *Mrgprd* in a *Trpv1*-negative jugular (*Prdm12*^+^) cluster (Fig 1, B; see also Fig E1, A, in this article’s Online Repository at www.jacionline.org). *Osmr*-expressing neurons were present in both jugular and nodose compartments. Nodose neurons also expressed *Lpar3* and *Osmr*, but in clusters lacking *Mrgprd*, *Mrgpra3*, or *Il31ra* (Fig 1, C). Instead, these nodose *Osmr*^+^ neurons coexpressed *Cckar1*, consistent with their proposed role as stomach-innervating neurons with intraganglionic laminar endings.^8^ Despite low cell numbers, jugular *Osmr*^+^ neurons coexpressed the NP2 and NP3 markers *Mrgpra3*, *Sst*, *Nppb*, and *Il31ra* (Fig 1, C) and could be further distinguished by the mutually exclusive expression of *Mrgpra3* and *Tac1* (Fig 1, C; see also Fig E1, B). These findings suggest that subsets of jugular VSNs share transcriptomic similarities with DRG-resident pruriceptor neurons.

**FIG 2.**
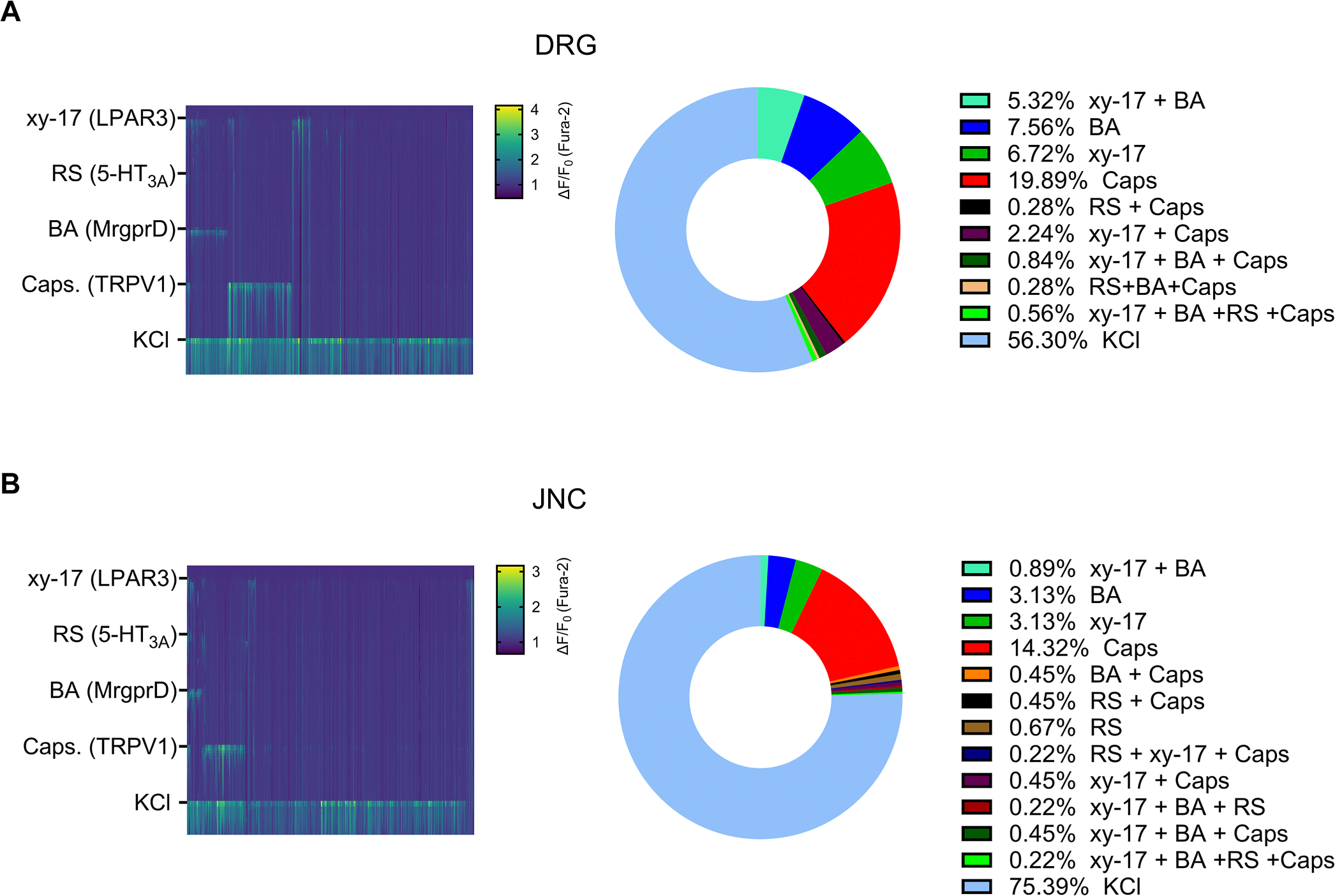
JNC neurons respond to agonists targeting NP1 neuron markers, MrgprD and LPAR3. **A** and **B,** Calcium influx in cultured neurons from the DRG (Fig 2, A) and JNC (Fig 2, B) was recorded in response to sequential treatments with the LPAR3 agonist xy-17 (10 μM, 60–70 seconds), the 5-HT3A agonist RS56812 (RS; 1 μM, 360–380 seconds), the MrgprD agonist β-alanine (BA; 1 mM, 660–680 seconds), the *Trpv1* agonist capsaicin (Caps; 1 μM, 960–970 seconds), and KCl (100 mM, 1260–1280 seconds). Data are presented as raster plots showing calcium flux responses or as doughnut charts indicating the proportion of neurons co-responsive (defined as an increase in Fura-2 acetoxymethyl intensity of >20% above baseline). N = 357 DRG neurons (Fig 2, A) and 461 JNC neurons (Fig 2, B).

To functionally validate these transcriptomic findings, we conducted real-time calcium imaging to test the responsiveness of LPAR3 and MrgprD in DRG and JNC neurons using their ligands xy-17 and β-alanine, respectively. Consistent with earlier studies, in DRG cultures, roughly 6% of analyzed neurons respond to xy-17 alone (LPAR3^+^), and approximately 7% were β-alanine–only responders (MrgprD^+^), whereas approximately 5% of the neurons are xy-17/β-alanine dual responders, populating roughly half of the total β-alanine–responsive (MrgprD^+^) neurons (Fig 2, A). Minimal overlap was observed between these neurons and TRPV1^+^ (capsaicin-responsive) cells, corroborating previous results.^37,38^ In cultured JNC neurons, a smaller fraction (~1%) responded to both β-alanine and xy-17, whereas approximately 3% responded only to β-alanine (Fig 2, B). Similar to DRG neurons, most β-alanine–responsive cells in the JNC did not respond to capsaicin (Fig 2, B).

**FIG 3.**
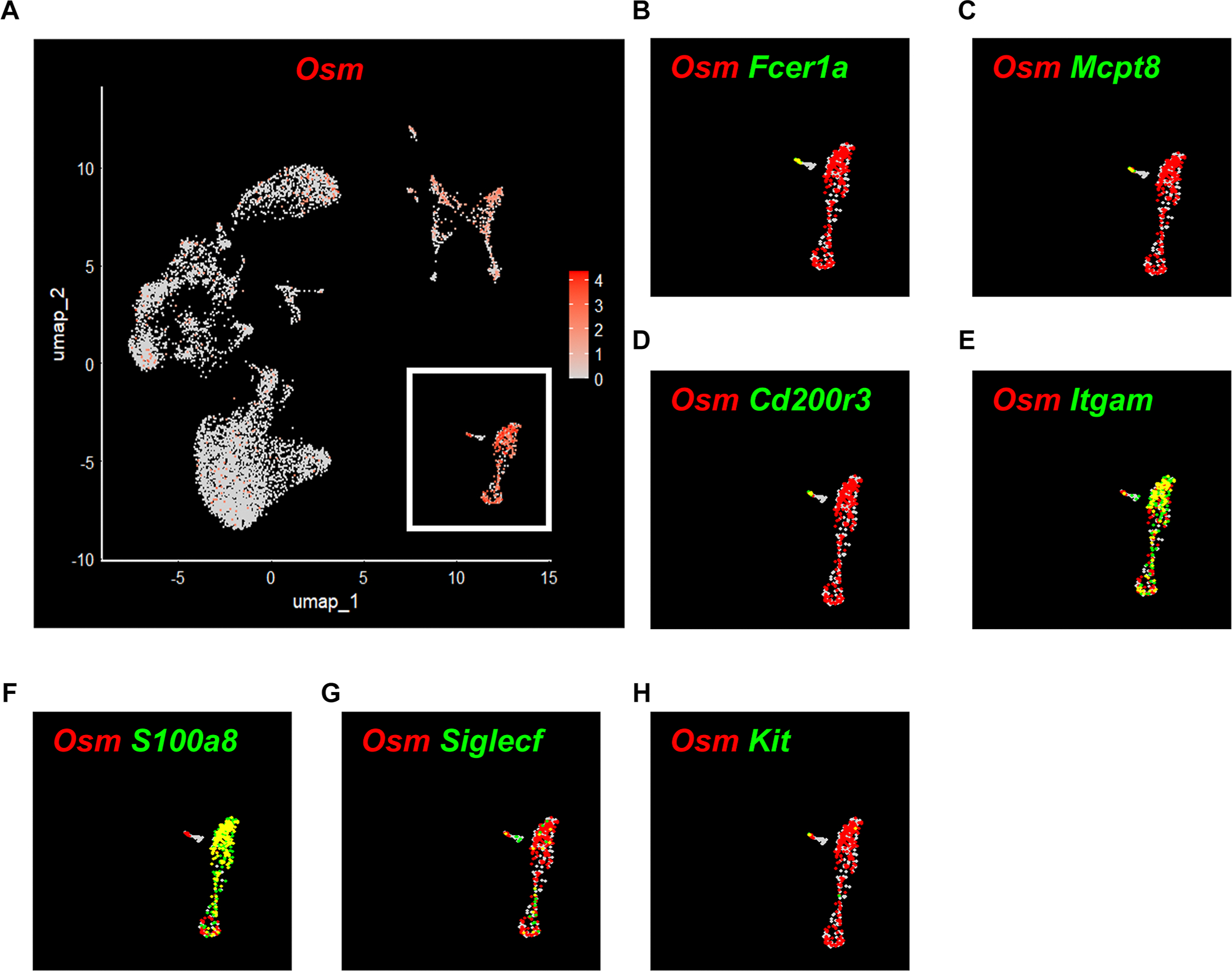
Lung basophils and neutrophils express *Osm* transcripts. **A-H,** UMAP plots generated from the data-base GSE278510^27^ feature pseudocolor highlighting of cells expressing *Osm* among all cells (Fig 3, A) and cells coexpressing markers for basophils—*Fcer1a* (Fig 3, B), *Mcpt8* (Fig 3, C), and *Cd200r3* (Fig 3, D); neutrophils—*Itgam* (Fig 3, E) and *S100a8* (Fig 3, F); eosinophils—*Itgam* (Fig 3, E) and *Siglecf* (Fig 3, G); and mast cells—*Fcer1a* (Fig 3, B) and *Kit* (Fig 3, H). The data are shown as colored dots, and the cells are labeled with nonzero values in counts per 10,000. The UMAP is derived from the database GSE278510, with experimental details outlined in Crosson et al.^27^

To investigate potential sources of OSM, the cytokine recognized by OSMR on NP2/NP3-like vagal neurons (Fig 1, A and C), we analyzed a mouse lung CD45^+^ scRNA-seq data set (GSE278510^27^). We identified 2 cell clusters highly expressing *Osm* (Fig 3, A): one coexpressing basophil markers, including *Fcer1a* (Fig 3, A and B), *Mcpt8* (Fig 3, C), and *Cd200r3* (Fig 3, D), and the other coexpressing neutrophil markers, *Itgam* (encoding CD11b; Fig 3, E) and *S100a8* (Fig 3, F). Compared with eosinophils (characterized by *Siglecf* [Fig 3, G] and *Itgam* [Fig 3, E] coexpression) or mast cells (defined by *Kit* [Fig 3, H] and *Fcer1a* [Fig 3, B]), *Osm* was more prominently enriched in basophils and some of the neutrophils. Supporting these findings, the ImmGen database also shows that lung basophils highly express the IL-6 family cytokines *Il6*, *Lif*, and *Osm*, as well as their signature cytokine *Il4* (see Fig E2, A,^39^ in this article’s Online Repository at www.jacionline.org). A human data set from CELLxGENE similarly indicates *OSM* expression in neutrophils and basophils (Fig E2, B).^40^

**FIG 4.**
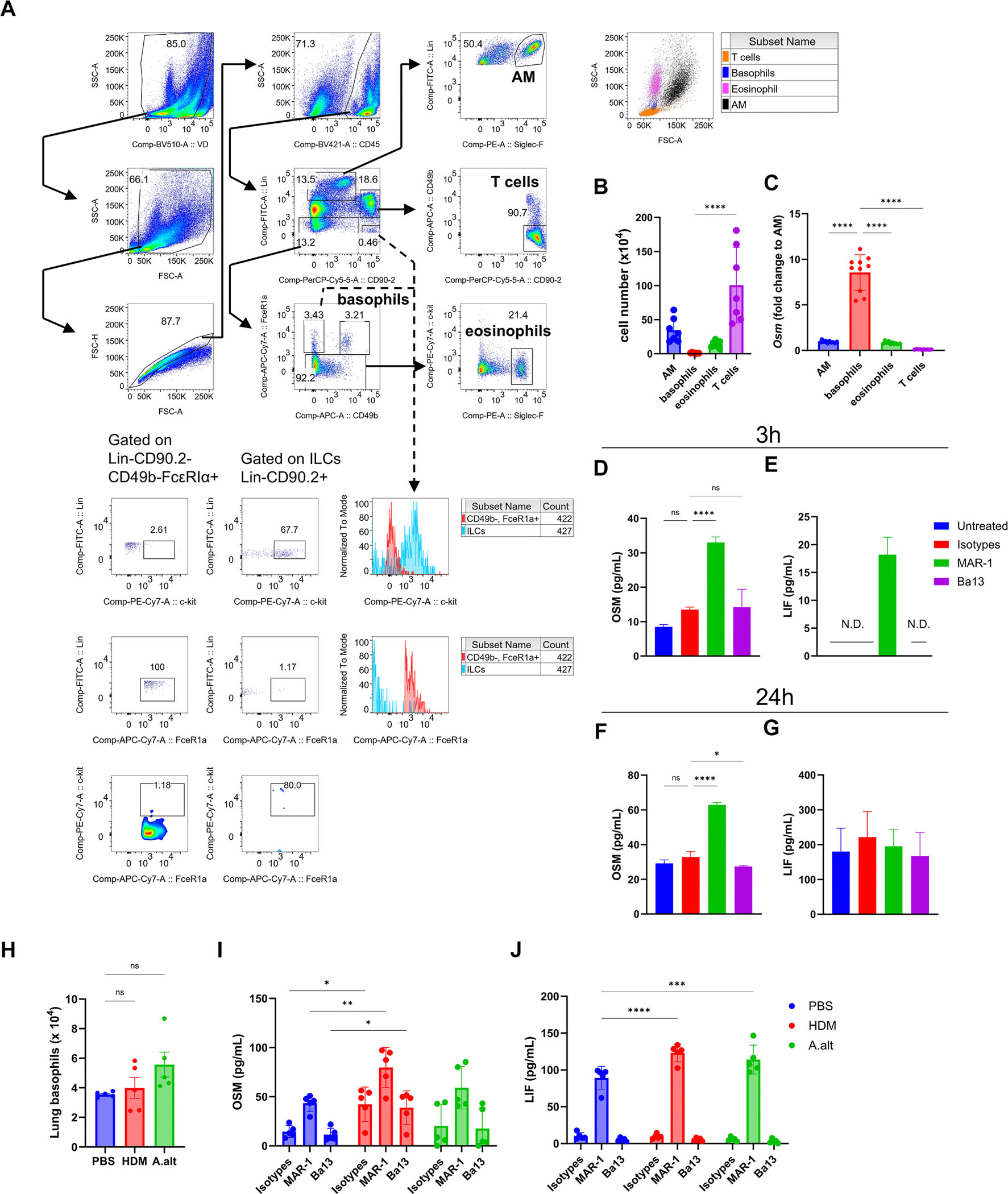
Lung basophils release OSM on FcεRIα engagement. **A-C,** Gating strategy eosinophils (Lin^−^ Siglec-F^+^ c-kit^−^ FcεRIα^−^), alveolar macrophages (AMs, Lin^+^ Siglec-F^+^), and T cells (Lin^+^ CD90.2^+^ CD49b^−^), basophils (Lin^−^ FcεRIα^+^ CD49b^+^), and c-kit–expressing cells under Lin^+^ CD90.2^+^ and Lin^+^ CD90.2^−^ CD49b^−^ Fc*ε*RI*α*^+^ gating. Counts (Fig 4, B) and transcript expression of *Osm* (Fig 4, C) in FACS-purified naive mouse lung eosinophils (Lin^−^ Siglec-F^+^ c-kit^−^ FcεRIα^−^), AMs (Lin^+^ Siglec-F^+^), T cells (Lin^+^ CD90.2^+^ CD49b^−^), and basophils (Lin^−^ FcεRIα^+^ CD49b^+^). **D-G,** 10^6^ lung cells/well were cultured from C57BL6 mice and stimulated with MAR-1 (1 μg/mL), Ba13 (1 μg/mL), or a mix of isotype control antibodies. Levels of OSM (Fig 4, D and F) and LIF (Fig 4, E and G) in the supernatant of cultured lung cells were assessed by ELISA 3 hours (Fig 4, D and E) and 24 hours (Fig 4, F and G) poststimulation. **H-J,** Six- to 10-week-old C57BL6 male and female mice were treated intranasally with PBS, HDM (20 μg/dose), or *A alternata* (100 μg/dose) from day 0 to day 4 and challenged on day 7 to day 9. Basophil numbers were assessed by flow cytometry (Fig 4, H), whereas OSM (Fig 4, I) and LIF (Fig 4, J) levels in the supernatant of cultured lung cells (10^6^ cells/well) were assessed by ELISA at 3 hours poststimulation with MAR-1 (1 μg/mL), Ba13 (1 μg/mL), or a mix of isotype control antibodies. Data are presented as FACS plots (Fig 4, A) or as mean ± SD (Fig 4, B–J). In Fig 4, B, data were pooled from 2 independent experiments, with 7 animals per group. In Fig 4, C, data were pooled from 3 independent experiments, with 10 animals per group. In Fig 4, D–G, representative data were from 2 independent experiments; lung cells were harvested and pooled from 3 animals, with 3 technical replicates per group. In Fig 4, H–J, representative data were from 2 independent experiments, with 5 animals per group and 1 technical replicate per animal. Statistical analyses were performed using 1-way ANOVA with the Dunnett test. Comparisons were made against all groups (Fig 4, B and C), isotype controls (Fig 4, D–G), or PBS control (Fig 4, H). *A. alt*, *A alternata*. *P ≤ .05; **P ≤ .01; ***P ≤ .001; ****P ≤ .0001.

Because OSM production by mouse basophils is not well characterized, we sorted lung basophils (Lin^−^ FcεRIα^+^ CD49b^+^) and then compared their *Osm* expression levels with those of eosinophils (Lin^−^ Siglec-F^+^ c-kit^−^ FcεRIα^−^), alveolar macrophages (Lin^+^ Siglec-F^+^), and T cells (Lin^+^ CD90.2^+^ CD49b^−^) (Fig 4, A). Although basophils were the least abundant population (Fig 4, B), they expressed the highest *Osm* levels (Fig 4, C). We then asked whether activated basophils release soluble OSM. Cultured lung cells were stimulated with antibodies that are known to activate basophils: MAR-1 against FcεRIα and Ba13 against CD200R3. OSM levels in the supernatant were measured at 3 and 24 hours. Stimulation with MAR-1, but not unstimulated or isotype-treated controls, significantly induced OSM release at both time points (Fig 4, D and F). MAR-1 also triggered the release of IL-4 (a prototypical basophil cytokine) at 3 and 24 hours (see Fig E3, A, in this article’s Online Repository at www.jacionline.org) and leukemia inhibitory factor (LIF) (another IL-6 family cytokine) at 3 hours (Fig 4, E), although LIF levels did not remain elevated at 24 hours (Fig 4, G). In contrast, Ba13 did not induce the release of these cytokines (Fig 4, D–G). We found that eosinophils in this lung preparation were FcεRIα^−^,^41^ and approximately 1% of Lin^−^ CD90.2^+^ FcεRIα^+^ cells were c-kit^+^ (Fig 4, A), indicating that MAR-1 activation primarily targeted basophils rather than eosinophils or mast cells.

We then examined whether OSM expression is altered in various mouse models of allergic airway inflammation—specifically, mice exposed to HDM (*Dermatophagoides pteronyssinus*) extract, *Alternaria alternata* medium, or OVA with or without fine particulate matter.^42^ Despite differences in granulocyte infiltration (Fig E3, B–E), lung *Osm* expression was consistently elevated in all allergic airway inflammation models (Fig E3, F and G). Although total basophil counts in homogenized lung tissue did not increase (Fig 4, H), cells from HDM-challenged mice released higher amounts of OSM both at baseline (isotype control) and 3 hours after MAR-1 stimulation (Fig 4, I). Moreover, although baseline LIF levels remained low in all groups, cells from HDM- and *A alternata*–treated mice showed greater LIF release following MAR-1 stimulation (Fig 4, J).

**FIG 5.**
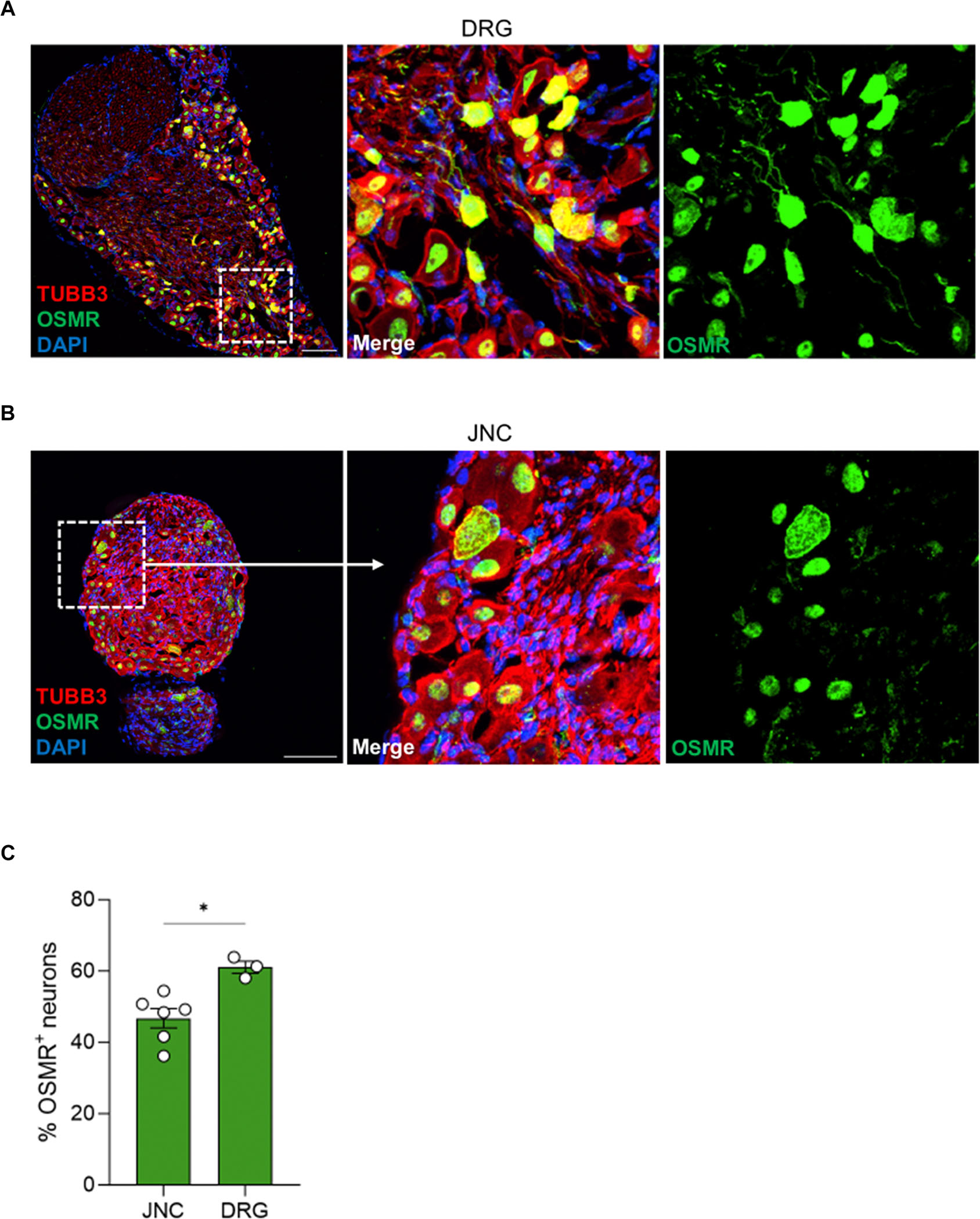
OSMR expression in VSNs visualized by immunofluorescence staining. **A** and **B,** Representative immunofluorescence images of β3-tubulin (TUBB3; *red*), OSMR (*green*), and nuclei (*blue*) in the DRG (Fig 5, A) and the JNC of naive 8-week-old male C57BL/6 mice (Fig 5, B). **C,** Quantification of OSMR^+^ neurons in the DRG and JNC indicates higher OSM expression in DRG neurons. For Fig 5, A–C, data are shown as representative immunofluorescence images (Fig 5, A and B) or mean ± SEM (Fig 5, C). The left panels show 20× magnification (scale bar = 100 μm) and the center and right panels provide zoomed-in views of the boxed regions. For Fig 5, A to C, n = 6 mice for JNC and n = 3 mice for DRG. Statistical analysis was performed using an unpaired Student *t* test (Fig 5, C). *P ≤ .05.

Given the presence of *Osmr* transcripts in VSNs, we performed immunofluorescent staining and confirmed OSMR expression in both DRG and JNC tissue sections from naive mice (Fig 5, A and B). In both DRG and JNC, OSMR immunoreactivity was detected primarily in neuron cell body (β3-tubulin^+^ cells) and in some nerve fibers (Fig 5, A and B). At baseline, the proportion of OSMR^+^ neurons was higher in DRG than in JNC (Fig 5, C).

**FIG 6.**
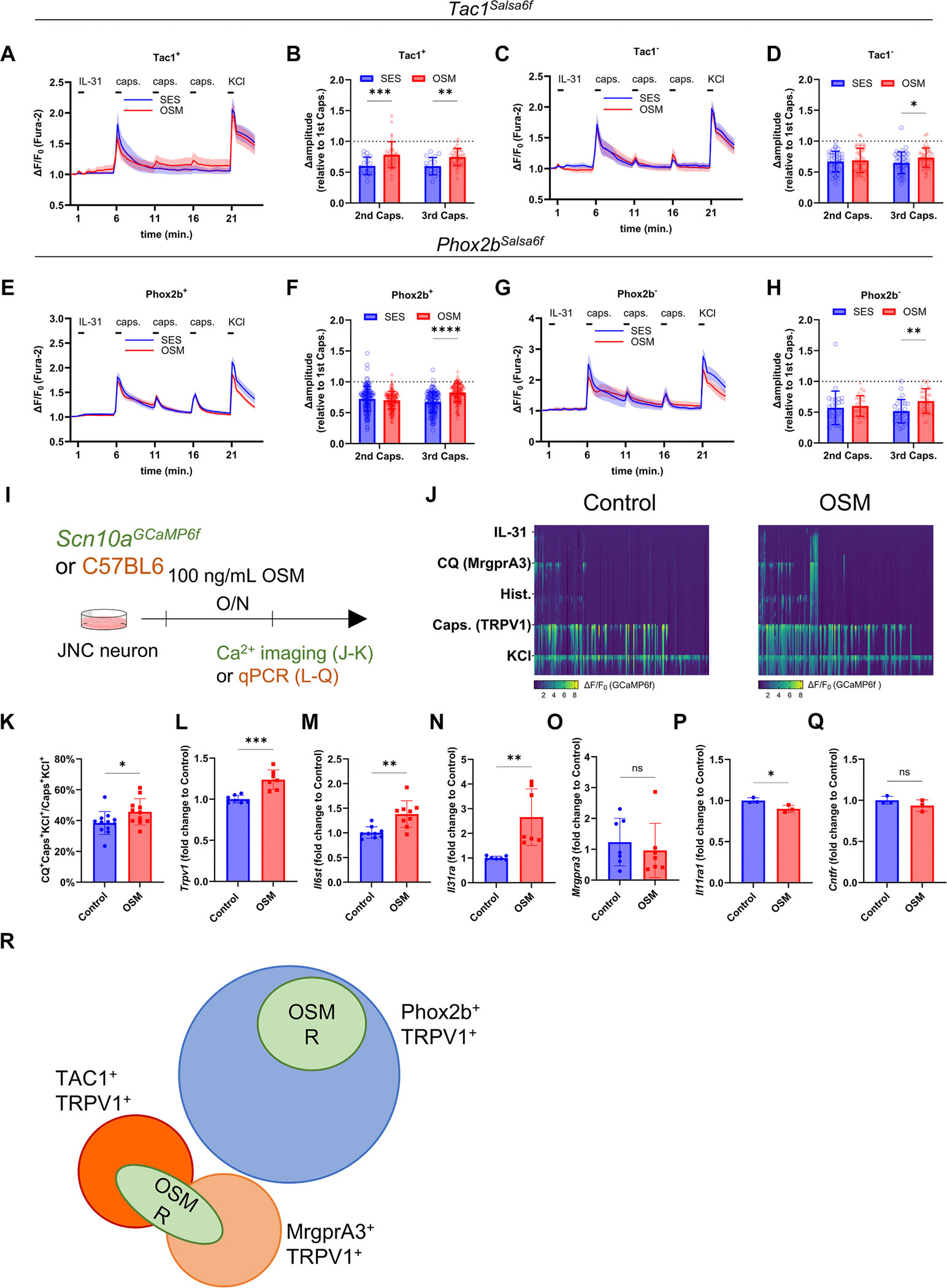
OSM prevents capsaicin-induced desensitization. **A-H,** Naive 6- to 12-week-old male and female *Tac1*^*Salsa6f*^ mice (Fig 6, A–D) or *Phox2b*^*Salsa6f*^ mice (Fig 6, E–H) were euthanized. JNC neurons were harvested, pooled, and cultured for 24 hours. After culturing, the cells were loaded with the calcium indicator Fura-2 AM and sequentially stimulated with IL-31 (100 ng/mL; from 60 to 120 seconds), capsaicin (Caps; 100 nM, at 360–370 seconds, 660–670 seconds, and 960–970 seconds), and KCl (100 mM; 1260–1280 seconds). Calcium flux was recorded throughout these stimulations. SES (*blue*) or 100 ng/mL OSM (*red*) was flown on the cells between the first and the second capsaicin exposure (420–660 seconds) (Fig 6, A, C, E, and G). The second and third capsaicin responses of Tac1^+^ (Fig 6, A and B; determined as tdTomato^+^), Tac1^−^ (Fig 6, C and D; determined as tdTomato^−^), Phox2b^+^ (Fig 6, E and F; determined as tdTomato^+^), and Phox2b^−^ (Fig 6, G and H; determined as tdTomato^−^) neurons were normalized to the initial response (at 360–370 seconds) of each neuron. **I-K,** Naive 6- to 12-week male and female *Scn10a*^*GCaMP6f*^ mice were euthanized, and JNC neurons were harvested, pooled, and cultured for 24 hours in the presence of vehicle or OSM (100 ng/mL). The cells were then sequentially stimulated with IL-31 (100 ng/mL, from 60 to 120 seconds), MrgprA3 agonist chloroquine (CQ; 1 mM, 360–375 seconds), histamine (Hist; 50 μM, 660–720 seconds), TRPV1 agonist capsaicin (Caps; 1 μM, 960–970 seconds), and KCl (40 mM, 1260–1285 seconds) and calcium flux was recorded. Proportions of CQ-responsive neurons among all capsaicin-responsive neurons (Fig 6, K). **L-Q,** Naive 6- to 12-week-old male and female C67BL6 mice were euthanized, and JNC neurons were harvested and cultured for 24 hours in the presence or absence of OSM (100 ng/mL). The cells were harvested, and gene expressions of *Trpv1* (Fig 6, L), *Il6st* (Fig 6, M), *Il31ra* (Fig 6, N), *Mrgpra3* (Fig 6, O), *Il11ra1* (Fig 6, P), and *Cntfr* (Fig 6, Q) were evaluated by qPCR. For Fig 6, A to H, JNC neurons were harvested from 10 mice per experiment, pooled into 10 dishes, and randomly assigned to either SES-treated or OSM-treated groups. One field of view was captured from each dish. **R,** A Venn diagram illustrating the relative overlap and distribution of OSMR^+^ neurons in the mouse JNC with other neuronal subtypes. Data are presented as mean ± 95% CI of the maximum Fura-2 AM (F/F_0_) fluorescence recorded every 15 seconds (Fig 6, A, C, E, and G), as mean ± SD (Fig 6, B, D, F, H, K–Q), as a heatmap (Fig 6, J), or as a Venn diagram (Fig 6, R). For Fig 6, A and B, analysis of n = 18 capsaicin-responsive Tac1^+^ SES-treated neurons and n = 38 OSM-treated neurons. For Fig 6, C and D, analysis of n = 40 capsaicin-responsive Tac1^−^ SES-treated neurons and n = 48 OSM-treated neurons. For Fig 6, E and F, analysis of n = 138 capsaicin-responsive Phox2b^+^ SES-treated neurons and n = 163 OSM-treated neurons. For Fig 6, G and H, analysis of n = 26 capsaicin-responsive Phox2b^−^ SES-treated neurons and n = 30 OSM-treated neurons. For Fig 6, I–K, JNC harvested from 10 mice per experiment was pooled into 10 dishes and randomly divided into untreated or OSM-treated groups. One field of view was captured from each dish. (Fig 6, J) Representative data from 3 independent experiments: n = 171 untreated neurons and n = 152 OSM-treated neurons. (Fig 6, K) Pooled data from 3 independent experiments: n = 12 fields of view for the control group and n = 11 for the OSM-treated group. In Fig 6, L to Q, for cell culture, 1 × 10^4^ cells/well were derived from 10 to 12 mice per experiment, with n = 3 to 9 technical replicates from a single experiment or pooled from 2 to 3 independent experiments. Data were analyzed by 2-way ANOVA with the Šidák test (Fig 6, B, D, F, and H) or by unpaired *t* test (Fig 6, K–Q). *P ≤ .05; **P ≤ .01; ***P ≤ .001; ****P ≤ .0001.

To investigate whether OSM modulates VSN activity, we first confirmed that OSM prevents TRPV1 desensitization in cultured DRG neurons (see Fig E4, A and B), consistent with previous studies.^11,24^ However, no such effect was observed in JNC cultures (Fig E4, C and D). Reasoning that the differential enrichment of OSMR between jugular and nodose neurons may mask the effect when analyzed together, we first focused on jugular neurons by using *Tac1*^*Salsa6f*^ reporter mice, given that TAC1^+^ TRPV1^+^ neurons are confined to the jugular compartment^4^ and some express *Osmr* (Fig 1, C; see also Fig E1, A and B). OSM exposure prevented desensitization to a second capsaicin application in *Tac1*^+^ (tdTomato^+^) neurons (Fig 6, A and B) but not in *Tac1*^−^ (Fig 6, C and D). In *Phox2b*^*Salsa6f*^ reporter mice, neither *Phox2b*^+^ (tdTomato^+^) nor *Phox2b*^−^ neurons were protected from desensitization to the second capsaicin exposure by OSM (Fig 6, E–H). In all cases, desensitization to the third capsaicin exposure was prevented by OSM (Fig 6, A–H).

Because jugular *Osmr*^+^ neurons can be subdivided into *Mrgpra3*^+^ and *Tac1*^+^ subsets, we tested whether OSM influences responses to the MrgprA3 agonist chloroquine. JNC neurons from *Scn10a*^*GCaMP6f*^ mice (Naᵥ1.8 lineage–driven GCaMP6f expression) were cultured, and those exposed overnight to OSM exhibited an increased proportion of chloroquine responders among capsaicin-responsive TRPV1^+^ neurons (Fig 6, I–K). At the population level, JNC cultures treated with OSM exhibited increased mRNA for *Trpv1*, *Il6st*, and *Il31ra* (Fig 6, L–N), but not for *Mrgpra3*, *Il11ra1*, or *Cntfr* (Fig 6, O–Q). Expression of NP3 markers—*Htr1f*, *Hrh1*, *Cysltr2*, *S1pr1*, *Sst*, *Nppb*, and *Fstl1*—as well as *Tac1* remained unchanged (Fig E4, F–M). Overall, these data suggest that OSM preferentially sensitizes neural crest-derived jugular and DRG neurons and, to a lesser extent, placode-derived nodose neurons (Fig 6, R).

**FIG 7.**
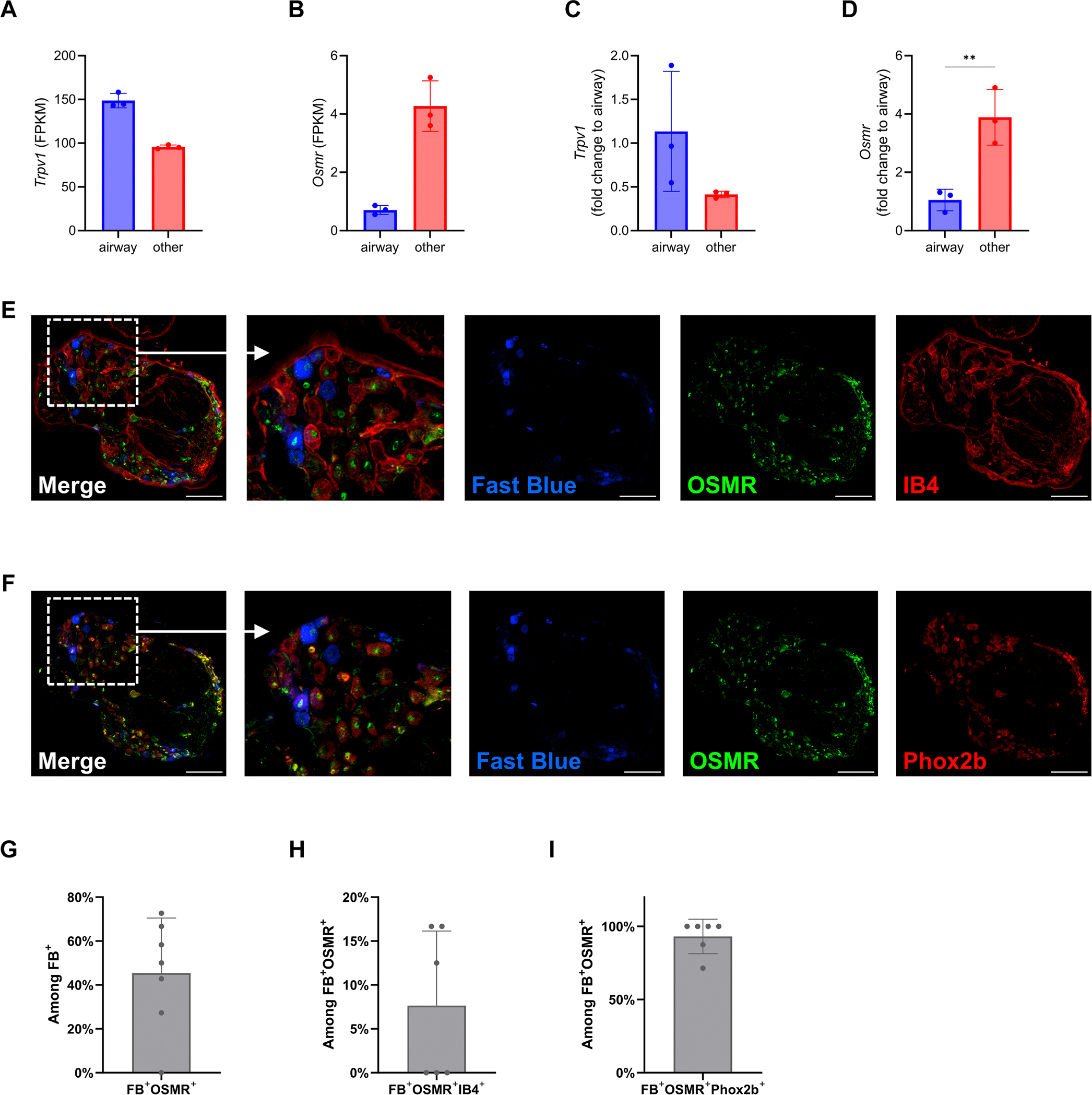
OSMR can be detected in airway-innervating VSNs. **A** and **B,** *In silico* analysis of bulk RNA sequencing (GSE223355^27^) comparing airway-innervating and non–airway-innervating Na_V_1.8^+^ neurons, showing transcript levels (FPKM) of *Trpv1* (Fig 7, A) and *Osmr* (Fig 7, B). **C** and **D,** Naive 6- to 12-week-old male and female *Scn10a*^*tdTomato*^ mice received an intranasal injection of DiD tracer (200 μM in 50 μL) to label airway-innervating neurons. Fourteen days later, JNCs were collected and dissociated, and airway-innervating (DiD^+^) and non–airway-innervating (DiD^−^) Na_V_1.8^+^ neurons (tdTomato^+^) were FACS-purified. RNA was extracted and qPCR performed to assess *Trpv1* (Fig 7, C) and *Osmr* (Fig 7, D) mRNA levels in each population. **E-I,** Naive 6- to 12-week-old male and female C57BL6 mice received an intranasal injection of Fast Blue (50 μL; 0.4 mM) to label airway-innervating neurons. Four days later, JNCs were collected, cryosectioned, and immunofluorescence-stained to visualize airway-innervating OSMR^+^ VSNs. Representative images of Fast Blue (FB; *blue*), OSMR (*green*), and IB4 binding (Fig 7, E; *red*) or Phox2b expression (Fig 7, F; *red*). Quantification of FB^+^OSMR^+^ (Fig 7, G), FB^+^OSMR^+^IB4^+^ (Fig 7, H), and FB^+^OSMR^+^Phox2b^+^ (Fig 7, I) percentages of cells among their respective parent populations. Data are presented as mean ± SD (Fig 7, A–D and G–I) or as representative images with 20× magnification (scale bar = 100 μm) (Fig 7, E and F). For Fig 7, A to D, n = 3 mice per group from 1 experiment. For Fig 7, G–I, n = 7 (*G*) or 6 (*H* and *I*) sections from 5 ganglia from 3 mice, representative of 2 independent experiments. Statistical analysis was performed using unpaired *t* tests (Fig 7, C and D) (**P ≤ .01). (Fig 7, A and B) The data were derived from the database GSE223355, with experimental details outlined in Crosson et al.^27^

We next asked whether *Osmr* is expressed by airway-innervating VSNs. We began with an *in silico* analysis of the published RNA-sequencing data set GSE223355, which includes airway-innervating and non–airway-innervating Na_V_1.8^+^ VSNs.^27^ This analysis revealed that both *Trpv1* and *Osmr* are detectable in the 2 neuronal populations (Fig 7, A and B). We then performed qPCR on FACS-purified Na_V_1.8^+^ VSNs to validate these findings. For this, *Scn10a*^*tdTomato*^ mice received intranasal injections of the lipophilic tracer dye DiD (200 μM) to label airway-innervating neurons. Fourteen days later, airway-innervating and non–airway-innervating Na_V_1.8^+^ VSNs were FACS-purified from dissociated JNC. Consistent with the *in silico* analysis (Fig 7, A and B) and previous work,^43^ *Trpv1* and *Osmr* were detected in airway-innervating VSNs, with higher expression observed in non–airway-innervating Na_V_1.8^+^ VSNs (Fig 7, C and D).

We next examined OSMR expression in airway-innervating VSNs by immunofluorescence staining of vagal ganglia. Sections were costained with isolectin B4 (IB4), which in the DRG marks nonpeptidergic populations—including MrgprD^+^ NP1 neurons^44^ and MrgprA3^+^ NP2 neurons,^45^ with minimal labeling of IL-31RA^+^ NP3 neurons^46^ and with anti-Phox2b to assess the jugular/nodose distribution of airway-innervating OSMR^+^ neurons. OSMR immunoreactivity was detected in Fast Blue–traced VSNs after intranasal injection, representing 45.41% ± 25.11% (mean ± SD) of total Fast Blue–traced VSNs (Fig 7, E–G), with partial overlap with IB4^+^ (Fig 7, E and H) and Phox2b^+^ VSNs (Fig 7, F and I). These findings indicate that OSMR-expressing, airway-innervating VSNs comprise a heterogeneous population. Notably, IB4 binding in OSMR^+^ neurons suggests similarities to nonpeptidergic DRG neurons.

**FIG 8.**
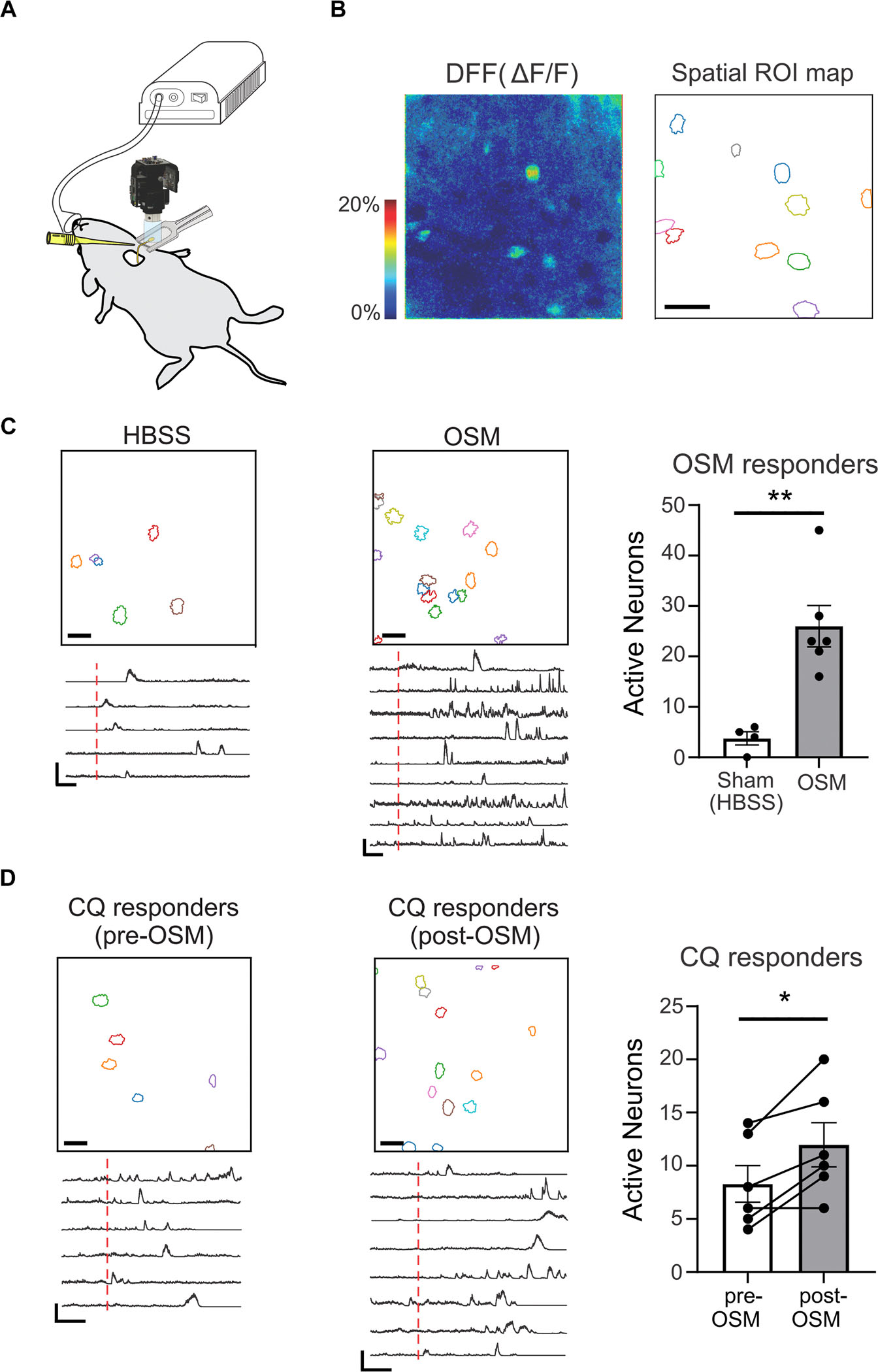
Airway delivery of OSM induces calcium influx and sensitizes CQ-responsive VSNs *in vivo*. **A,** Schematic of the experimental setup for vagal ganglion calcium imaging, illustrating the Miniscope, nebulizer for intranasal administration, and direct vagus nerve application. **B,** Changes in fluorescence intensity were recorded, and individually activated neurons were labeled on an ROI map. **C,** *left* and *center*, Representative ROI maps and calcium fluorescence traces of active neurons during intranasal delivery of HBSS or 100 ng/mL OSM, showing significantly more neurons activated by OSM. *Right*, Quantification of the number of active neurons under OSM exposure. **D,** *left* and *center*, Representative ROI maps and calcium fluorescence traces of neurons responding to 1 mM CQ applied to the vagus nerve before and after intranasal OSM exposure. *Right*, Quantification of CQ-responsive neurons pre- and post-OSM. *CQ*, Chloroquine; *ROI*, region of interest. Data are presented as schematic (Fig 8, A), representative calcium microscopy images (Fig 8, B), representative ROI maps (Fig 8, C and D), or mean ± SEM (Fig 8, C and D). “L” scale bars for all traces (Fig 8, C and D): (y) 60 ΔF/F and (x) 30 s. Scale bars in ROI maps: 50 μm. Analyses were performed using 4 mice (HBSS), 6 mice (OSM) (Fig 8, C), or 6 mice (Fig 8, D) per group. Statistical significance was determined by unpaired Student *t* test. *P < .05; **P < .005.

To further explore this similarity, we tested whether airway-delivered OSM alters VSN responses to the MrgprA3 agonist chloroquine. To examine OSM’s effects on lung-innervating VSNs *in vivo*, we performed live calcium imaging of vagal ganglia of *Slc17a6*^*GCaMP6f*^ (Vglut2-lineage GCaMP6f-labeled neurons) mice during intranasal OSM administration.^30,31^ Using a nebulizer, we delivered OSM into the airway and continuously monitored neuronal activity during OSM exposure and subsequent chloroquine application (Fig 8, A and B). Intranasal OSM significantly increased the number of activated vagal neurons compared with sham stimulation using HBSS (Fig 8, C), indicating direct activation of VSNs by airway-delivered OSM. Moreover, chloroquine-evoked responses were enhanced 5 minutes after OSM administration (Fig 8, D), suggesting that OSM can sensitize MrgprA3-dependent activation of VSNs. In summary, our findings characterize MrgprD- and OSMR-expressing VSNs within the mouse JNC. These neurons respond to xy-17/β-alanine and are sensitized by OSM—a cytokine produced by lung basophils during allergic airway inflammation.

## Discussion

Although the expression of pruritogen receptors in VSNs has been revealed by scRNA-seq, the existence and overall role of these neurons remain to be fully elucidated. Here, we used a combination of lineage reporter lines to investigate subtypes of VSNs derived from either the placode or the neural crest, focusing on their ability to detect pruritogenic mediators. Specifically, we characterized the responsiveness of these VSN subsets to LPA, β-alanine, chloroquine, and the cytokine OSM. We discovered that OSM is produced by lung-resident basophils, and its release is not only triggered by FcεRIα engagement but also elevated after HDM extract stimulation. Furthermore, OSM prevents the desensitization of capsaicin response and increases chloroquine response in Tac1^+^TRPV1^+^ and MrgprA3^+^ VSNs likely derived from jugular ganglia. Collectively, these findings suggest a novel basophil-neuron axis that may exacerbate type I hypersensitivity diseases such as allergic asthma.

### Mrgpr family proteins as jugular neuron markers

In mice, the jugular and nodose ganglia are anatomically fused. Although agonists of P2X_2/3_^47^ and protease-activated receptor 1^48^ can selectively activate nodose neurons, a specific activator for jugular neurons remains to be better defined. The expression of conventional nociceptor markers such as TRPV1, TRPA1, or TAC1 is not exclusive to jugular VSNs. However, scRNA-seq data indicate that *Mrgpra3* and *MrgprD* are uniquely expressed in jugular VSNs. Later findings not only confirmed this with *in situ* hybridization, but also showed that reporter-marked MrgprC11 fibers were found in mouse tracheal,^49^ providing a new way to study airway jugular neuron activity.

### Lysophosphatidic acid

LPA can be synthesized by extracellular autotaxin.^50^ Intracellular LPA induces itch by engaging receptors such as LPAR5, TRPV1, or TRPA1.^51^ Elevated LPA levels have been reported in BALF from allergic or asthmatic patients after airway allergen challenge,^52^ promoting T_H_2-type inflammation and supporting epithelial barrier function via eosinophil and epithelial cell interactions.^53^ When administered intravenously, LPA activates the carotid body, stimulating the vagus nerve and causing bronchoconstriction,^54^ a response exacerbated in an OVA-induced rat asthma model.^55^ Our data showing the xy-17–induced calcium flux in cultured vagal neurons suggest that LPAR3-expressing VSNs populated half of the MrgprD^+^ (β-alanine–respondent) neurons. Whether these neurons respond to LPA in concert with the carotid body during asthma attacks remains unknown.

### Oncostatin M

OSM, a member of the IL-6 cytokine family, typically signals through heterodimeric receptors composed of OSMR and gp130, although in rats and humans it can also bind the LIF receptor.^56,57^ Elevated OSM levels have been reported in idiopathic pulmonary fibrosis,^58^ chronic obstructive pulmonary disease,^59^ asthma with incompletely reversible airflow limitation,^60^ and coronavirus disease 2019.^61,62^ Although some studies have proposed neutrophils,^63^ monocytes, or T cells^11^ as the primary sources of OSM, scRNA-seq analyses indicate that basophils express high levels of *Osm*. Here, we confirmed that mouse basophils express *Osm* mRNA and release OSM on FcεRIα engagement. Because mouse eosinophils generally lack FcεRIα expression and mast cells are scarce in the lung parenchyma, FcεRIα-mediated activation predominantly targets basophils. In human data sets (eg, CELLxGENE), *OSM* expression is attributed to basophils and neutrophils, consistent with our *in silico* analysis of the scRNA-seq data set GSE278510.^27^

### Neuron-basophil crosstalk

The correlation between basophil activation and disease severity underscores the critical role basophils play in allergy pathophysiology.^64^ In mouse models, acute allergen-induced itch is usually mediated by a mast cell–histamine mechanism. However, in atopic dermatitis, this mechanism becomes redundant, and a basophil-leukotriene pathway emerges as the primary mediator of itch,^12^ indicating a novel basophil-neuronal circuit in certain inflammatory conditions. Our observation of enhanced OSM and LIF levels in allergic airway inflammation suggests that a similar basophil-neuron axis may be active in the lung.

Traditionally, basophils have been regarded as circulating cells recruited to the lungs during T_H_2-mediated inflammation.^65,66^ However, Cohen et al^18^ demonstrated that basophils are present in the lungs from birth, where they cooperate with epithelial cells to promote alveolar macrophage maturation. Despite their relatively small numbers (~20,000 cells), we found that lung-resident basophils produce high levels of IL-4 and IL-6 family cytokines and can rapidly secrete them on FcεRIα ligation, even in naive mice. Given (1) the absence of a significant basophil increase in allergic mice and (2) the ability of naive basophils to release cytokines, we propose that lung-resident basophils are the principal source of OSM in allergic airway disease. Because LIF receptors are expressed on a broader range of sensory neurons than OSMR, OSM’s dual affinity for LIF receptor in humans may allow it to act on neuronal populations beyond OSMR^+^ VSNs. Furthermore, we observed that LIF production can be induced by FcεRIα engagement, suggesting that basophils may modulate multiple VSN subtypes.

### OSM maintains VSN subset activity

Capsaicin, the pungent compound in chili peppers, is a well-known agonist of TRPV1 channels expressed by nociceptive sensory neurons.^67^ Prolonged or repeated exposure to capsaicin induces TRPV1 desensitization—an adaptive process involving calcium-dependent dephosphorylation, receptor internalization, and reduced channel responsiveness.^68,69^ Although this desensitization protects neurons from overstimulation, agents that prevent TRPV1 desensitization can sustain nociceptor activity and exacerbate allergic inflammation.^70–73^ OSM has previously been implicated in chronic itch in mouse models,^11^ and later work linked OSM-OSMR signaling to neuropathic pain phenotypes in human DRG.^74^

Protein kinase A and protein kinase C are key regulators of TRPV1 responsiveness. Nodose *Osmr*^+^ neurons coexpress *Prkaca* (encoding the protein kinase A catalytic subunit α) (Fig E1, C), whereas jugular *Osmr*^+^ neurons coexpress *Prkca* (encoding protein kinase C α) (Fig E1, D). In addition, *Prkcq* (encoding protein kinase C θ) is enriched in jugular *Mrgprd*^+^ neurons (Fig E1, E and F). These findings suggest that OSM may prevent capsaicin desensitization through distinct mechanisms across different *Osmr*^+^ vagal neuron subsets.

## Conclusions

Our data demonstrate that *Osmr*-expressing VSNs can be modulated by basophil-derived OSM, a process that may contribute to the exacerbation of neurogenic symptoms in allergic asthma and other type I hypersensitivity disorders.

## Supplementary Material

1

## Key messages

- Findings in mice reveal OSMR-expressing VSNs and highlight the release of OSM by activated lung basophils.
- These results suggest a previously unrecognized basophil-neuron axis that may contribute to the pathogenesis of allergic asthma.

## Abbreviations used

BALF: Bronchoalveolar lavage fluid
DMEM: Dulbecco modified Eagle medium
DRG: Dorsal root ganglia
FACS: Fluorescence-activated cell sorting
HDM: House dust mite
IB4: Isolectin B4
JNC: Jugular-nodose complex
LIF: Leukemia inhibitory factor
LPA: Lysophosphatidic acid
LPAR: LPA receptor
Mrgpr: Mas-related G protein–coupled receptor
OSM: Oncostatin M
OSMR: OSM receptor β
OVA: Ovalbumin
Phox2b: Paired-like homeobox 2B
Prdm12: PR domain zinc finger protein 12
qPCR: Quantitative PCR
scRNA-seq: Single-cell RNA sequencing
SES: Standard external solution
SsT: Somatostatin
TRP: Transient receptor potential
UMAP: Uniform Manifold Approximation and Projection
VSN: Vagal sensory neuron

## References

[1] Mazzone SB, Undem BJ. Vagal afferent innervation of the airways in health and disease. Physiol Rev 2016;96:975–1024. 27279650 10.1152/physrev.00039.2015 PMC4982036

[2] Crosson T, Talbot S. Anatomical differences in nociceptor neurons sensitivity. Bio-electron Med 2022;8:7. 10.1186/s42234-022-00088-w PMC8985299 35382899

[3] Crosson T, Roversi K, Balood M, Othman R, Ahmadi M, Wang JC, Profiling of how nociceptor neurons detect danger—new and old foes. J Intern Med 2019;286:268–89. 31282104 10.1111/joim.12957

[4] Taylor-Clark TE. Molecular identity, anatomy, gene expression and function of neural crest vs. placode-derived nociceptors in the lower airways. Neurosci Lett 2021;742:135505. 33197519 10.1016/j.neulet.2020.135505 PMC7779741

[5] Vermeiren S, Bellefroid EJ, Desiderio S. Vertebrate sensory ganglia: common and divergent features of the transcriptional programs generating their functional specialization. Front Cell Dev Biol 2020;8:587699. 33195244 10.3389/fcell.2020.587699 PMC7649826

[6] Gu Q, Lee L-Y. TRP channels in airway sensory nerves. Neurosci Lett 2021;748:135719. 33587987 10.1016/j.neulet.2021.135719 PMC7988689

[7] Kollarik M, Ru F, Brozmanova M. Vagal afferent nerves with the properties of nociceptors. Auton Neurosci 2010;153:12–20. 19751993 10.1016/j.autneu.2009.08.001 PMC2818152

[8] Kupari J, Häring M, Agirre E, Castelo-Branco G, Ernfors P. An atlas of vagal sensory neurons and their molecular specialization. Cell Rep 2019;27:2508–23.e4. 31116992 10.1016/j.celrep.2019.04.096 PMC6533201

[9] Kupari J, Ernfors P. Molecular taxonomy of nociceptors and pruriceptors. Pain 2023;164:1245. 36718807 10.1097/j.pain.0000000000002831 PMC10184562

[10] Tominaga M, Takamori K. Peripheral itch sensitization in atopic dermatitis. Allergol Int 2022;71:265–77. 35624035 10.1016/j.alit.2022.04.003

[11] Tseng P-Y, Hoon MA. Oncostatin M can sensitize sensory neurons in inflammatory pruritus. Sci Transl Med 2021;13:eabe3037. 34757808 10.1126/scitranslmed.abe3037 PMC9595590

[12] Wang F, Trier AM, Li F, Kim S, Chen Z, Chai JN, A basophil-neuronal axis promotes itch. Cell 2021;184:422–40. 33450207 10.1016/j.cell.2020.12.033 PMC7878015

[13] Hashimoto T, Rosen JD, Sanders KM, Yosipovitch G. Possible roles of basophils in chronic itch. Exp Dermatol 2019;28:1373–9. 29894005 10.1111/exd.13705

[14] Gupta K, Harvima IT. Mast cell-neural interactions contribute to pain and itch. Immunol Rev 2018;282:168–87. 29431216 10.1111/imr.12622 PMC5812374

[15] Stone KD, Prussin C, Metcalfe DD. IgE, mast cells, basophils, and eosinophils. J Allergy Clin Immunol 2010;125:S73–80. 20176269 10.1016/j.jaci.2009.11.017 PMC2847274

[16] Flora M, Perna F, Abbadessa S, Garziano F, Maffucci R, Maniscalco M, Basophil activation test for *Staphylococcus aureus* enterotoxins in severe asthmatic patients. Clin Exp Allergy 2021;51:536–45. 33131112 10.1111/cea.13772

[17] Boita M, Heffler E, Omedè P, Bellocchia M, Bussolino C, Solidoro P, Basophil membrane expression of epithelial cytokine receptors in patients with severe asthma. Int Arch Allergy Immunol 2018;175:171–6. 29402810 10.1159/000486314

[18] Cohen M, Giladi A, Gorki A-D, Solodkin DG, Zada M, Hladik A, Lung single-cell signaling interaction map reveals basophil role in macrophage imprinting. Cell 2018;175:1031–44.e18. 30318149 10.1016/j.cell.2018.09.009

[19] Schaum N, Karkanias J, Neff NF, May AP, Quake SR, Wyss-Coray T, Tabula Muris Consortium, Single-cell transcriptomics of 20 mouse organs creates a Tabula Muris. Nature 2018;562:367–72. 30283141 10.1038/s41586-018-0590-4 PMC6642641

[20] Travaglini KJ, Nabhan AN, Penland L, Sinha R, Gillich A, Sit RV, A molecular cell atlas of the human lung from single-cell RNA sequencing. Nature 2020;587:619–25. 33208946 10.1038/s41586-020-2922-4 PMC7704697

[21] Nadif R, Henny J, Tsiavia T, Ribet C, Goldberg M, Zins M, Blood basophils, nocturnal cough and asthma in the Constances cohort. Eur Respir J 2023;62(Suppl 67)PA4430.

[22] Kauffmann F, Neukirch F, Annesi I, Korobaeff M, Doré MF, Lellouch J. Relation of perceived nasal and bronchial hyperresponsiveness to FEV_1_, basophil counts, and methacholine response. Thorax 1988;43:456–61. 3420556 10.1136/thx.43.6.456 PMC461310

[23] Leyva-Castillo J-M, Vega-Mendoza D, Strakosha M, Deng L, Choi S, Miyake K, Basophils are important for the development of allergic skin inflammation. J Allergy Clin Immunol 2024;153:1344–54.e5. 38336257 10.1016/j.jaci.2024.01.022 PMC11070311

[24] Langeslag M, Constantin CE, Andratsch M, Quarta S, Mair N, Kress M. Oncostatin M induces heat hypersensitivity by gp130-dependent sensitization of TRPV1 in sensory neurons. Mol Pain 2011;7:102. 22196363 10.1186/1744-8069-7-102 PMC3275481

[25] Tochitsky I, Jo S, Andrews N, Kotoda M, Doyle B, Shim J, Inhibition of inflammatory pain and cough by a novel charged sodium channel blocker. Br J Pharmacol 2021;178:3905–23. 33988876 10.1111/bph.15531 PMC8643097

[26] Zhao Q, Yu CD, Wang R, Xu QJ, Dai Pra R, Zhang L, A multidimensional coding architecture of the vagal interoceptive system. Nature 2022;603:878–84. 35296859 10.1038/s41586-022-04515-5 PMC8967724

[27] Crosson T, Bhat S, Wang JC, Salaun C, Fontaine E, Roversi K, Cytokines reprogram airway sensory neurons in asthma. Cell Rep 2024;43:115045. 39661516 10.1016/j.celrep.2024.115045

[28] Perner C, Sokol CL. Protocol for dissection and culture of murine dorsal root ganglia neurons to study neuropeptide release. STAR Protoc 2021;2:100333. 33615276 10.1016/j.xpro.2021.100333 PMC7876630

[29] Wang J-C, Crosson T, Talbot S. Analysis of airway vagal neurons. Methods Mol Biol 2022;2506:297–314. 35771480 10.1007/978-1-0716-2364-0_21

[30] Huerta TS, Haider B, Adamovich-Zeitlin R, Chen AC, Chaudhry S, Zanos TP, Calcium imaging and analysis of the jugular-nodose ganglia enables identification of distinct vagal sensory neuron subsets. J Neural Eng 2023;20:026014. 10.1088/1741-2552/acbe1e PMC10790314 36920156

[31] Huerta TS, Chen AC, Chaudhry S, Tynan A, Morgan T, Park K, Neural representation of cytokines by vagal sensory neurons. Nat Commun 2025;16:3840. 40268933 10.1038/s41467-025-59248-6 PMC12019601

[32] Cai DJ, Aharoni D, Shuman T, Shobe J, Biane J, Song W, A shared neural ensemble links distinct contextual memories encoded close in time. Nature 2016;534:115–8. 27251287 10.1038/nature17955 PMC5063500

[33] Giovannucci A, Friedrich J, Gunn P, Kalfon J, Brown BL, Koay SA, CaImAn an open source tool for scalable calcium imaging data analysis. Elife 2019;8:e38173. 30652683 10.7554/eLife.38173 PMC6342523

[34] Roversi K, Tabatabaei M, Desjardins-Lecavalier N, Balood M, Crosson T, Costantino S, Nanophotonics enable targeted photothermal silencing of nociceptor neurons. Small 2022;18:e2103364. 35195345 10.1002/smll.202103364

[35] Lin JC, Roy JP, Verreault J, Talbot S, Cote F, Couture R, An ex vivo approach to the differential parenchymal responses induced by cigarette whole smoke and its vapor phase. Toxicology 2012;293:125–31. 22266391 10.1016/j.tox.2012.01.004

[36] Talbot S, Lin JC, Lahjouji K, Roy JP, Senecal J, Morin A, Cigarette smokeinduced kinin B1 receptor promotes NADPH oxidase activity in cultured human alveolar epithelial cells. Peptides 2011;32:1447–56. 21600945 10.1016/j.peptides.2011.05.005

[37] George DS, Jayaraj ND, Pacifico P, Ren D, Sriram N, Miller RE, The Masrelated G protein-coupled receptor d (Mrgprd) mediates pain hypersensitivity in painful diabetic neuropathy. Pain 2022;10:1097. 10.1097/j.pain.0000000000003120 PMC11017747 38147415

[38] Liu Q, Dong X. The role of the Mrgpr receptor family in itch. Handb Exp Pharmacol 2015;226:71–88. 25861775 10.1007/978-3-662-44605-8_5

[39] Gal-Oz ST, Maier B, Yoshida H, Seddu K, Elbaz N, Czysz C, ImmGen report: sexual dimorphism in the immune system transcriptome. Nat Commun 2019;10:4295. 31541153 10.1038/s41467-019-12348-6 PMC6754408

[40] Biology CS-C, Abdulla S, Aevermann B, Assis P, Badajoz S, Bell SM, CZ CELLxGENE Discover: a single-cell data platform for scalable exploration, analysis and modeling of aggregated data. bioRxiv preprint November 2, 2023. 10.1101/2023.10.30.563174. PMC11701654 39607691

[41] De Andres B, Rakasz E, Hagen M, McCormik ML, Mueller AL, Elliot D, Lack of Fc-ε receptors on murine eosinophils: implications for the functional significance of elevated IgE and eosinophils in parasitic infections. Blood 1997;89:3826–36. 9160690

[42] Wang J-C, Crosson T, Nikpoor AR, Gupta S, Rafei M, Talbot S. Nociceptor neurons control pollution-mediated neutrophilic asthma. bioRxiv preprint June 21, 2025. 10.1101/2024.08.22.609202. PMC13030891 41891831

[43] Liu Y, Diaz de Arce AJ, Krasnow MA. Molecular, anatomical, and functional organization of lung interoceptors. bioRxiv preprint November 13, 2021. 10.1101/2021.11.10.468116.

[44] Reynders A, Moqrich A. Analysis of cutaneous MRGPRD free nerve endings and C-LTMRs transcriptomes by RNA-sequencing. Genom Data 2015;5:132–5. 26484241 10.1016/j.gdata.2015.05.022 PMC4583636

[45] Han L, Ma C, Liu Q, Weng H-J, Cui Y, Tang Z, A subpopulation of nociceptors specifically linked to itch. Nat Neurosci 2013;16:174–82. 23263443 10.1038/nn.3289 PMC3557753

[46] Cevikbas F, Wang X, Akiyama T, Kempkes C, Savinko T, Antal A, A sensory neuron-expressed IL-31 receptor mediates T helper cell-dependent itch: involvement of TRPV1 and TRPA1. J Allergy Clin Immunol 2014;133:448–60.e7. 24373353 10.1016/j.jaci.2013.10.048 PMC3960328

[47] Kwong K, Kollarik M, Nassenstein C, Ru F, Undem BJ. P2X2 receptors differentiate placodal vs. neural crest C-fiber phenotypes innervating guinea pig lungs and esophagus. Am J Physiol Lung Cell Mol Physiol 2008;295:L858–65. 18689601 10.1152/ajplung.90360.2008 PMC2584877

[48] Sun H, Meeker S, Undem BJ. Role of TRP channels in Gq-coupled protease-activated receptor 1-mediated activation of mouse nodose pulmonary C-fibers. Am J Physiol Lung Cell Mol Physiol 2020;318:L192–9. 31664854 10.1152/ajplung.00301.2019 PMC6985869

[49] Xing Y, Nho Y, Lawson K, Zhu Y, Ellison AE, Chang MY, MrgprC11+ jugular neurons control airway hyperresponsiveness in allergic airway inflammation. Am J Respir Cell Mol Biol 2025;72:393–407. 39405479 10.1165/rcmb.2024-0153OC PMC12005045

[50] Geraldo LHM, Spohr TCLS, Amaral RFD, Fonseca ACCD, Garcia C, Mendes FA, Role of lysophosphatidic acid and its receptors in health and disease: novel therapeutic strategies. Signal Transduct Target Ther 2021;6:45. 33526777 10.1038/s41392-020-00367-5 PMC7851145

[51] Kittaka H, Uchida K, Fukuta N, Tominaga M. Lysophosphatidic acid-induced itch is mediated by signalling of LPA5 receptor, phospholipase D and TRPA1/TRPV1. J Physiol 2017;595:2681–98. 28176353 10.1113/JP273961 PMC5390871

[52] Georas SN, Berdyshev E, Hubbard W, Gorshkova IA, Usatyuk PV, Saatian B, Lysophosphatidic acid is detectable in human bronchoalveolar lavage fluids at baseline and increased after segmental allergen challenge. Clin Exp Allergy 2007;37:311–22. 17359381 10.1111/j.1365-2222.2006.02626.x

[53] Park GY, Lee YG, Berdyshev E, Nyenhuis S, Du J, Fu P, Autotaxin production of lysophosphatidic acid mediates allergic asthmatic inflammation. Am J Respir Crit Care Med 2013;188:928–40. 24050723 10.1164/rccm.201306-1014OC PMC3826286

[54] Jendzjowsky NG, Roy A, Barioni NO, Kelly MM, Green FH, Wyatt CN, Preventing acute asthmatic symptoms by targeting a neuronal mechanism involving carotid body lysophosphatidic acid receptors. Nat Commun 2018;9:4030. 30279412 10.1038/s41467-018-06189-y PMC6168495

[55] Jendzjowsky NG, Roy A, Wilson RJA. Asthmatic allergen inhalation sensitises carotid bodies to lysophosphatidic acid. J Neuroinflamm 2021;18:1–8. 10.1186/s12974-021-02241-9 PMC8408927 34465362

[56] Houben E, Hellings N, Broux B. Oncostatin M, an underestimated player in the central nervous system. Front Immunol 2019;10:1165. 31191538 10.3389/fimmu.2019.01165 PMC6549448

[57] Walker EC, Johnson RW, Hu Y, Brennan HJ, Poulton IJ, Zhang J-G, Murine oncostatin M acts via leukemia inhibitory factor receptor to phosphorylate signal transducer and activator of transcription 3 (STAT3) but not STAT1, an effect that protects bone mass. J Biol Chem 2016;291:21703–16. 27539849 10.1074/jbc.M116.748483 PMC5076839

[58] Majewski S, Zhou X, Mikowska-Dymanowska J, Biaas AJ, Piotrowski WJ, Malinovschi A. Proteomic profiling of peripheral blood and bronchoalveolar lavage fluid in interstitial lung diseases: an explorative study. ERJ Open Res 2021;7:00489, 2020. 33816595 10.1183/23120541.00489-2020 PMC8005592

[59] Vanfleteren LEGW, Weidner J, Franssen FME, Gaffron S, Reynaert NL, Wouters EFM, Biomarker-based clustering of patients with chronic obstructive pulmonary disease. ERJ Open Res 2023;9:00301, 2022. 36755966 10.1183/23120541.00301-2022 PMC9900445

[60] Simpson JL, Baines KJ, Boyle MJ, Scott RJ, Gibson PG. Oncostatin M (OSM) is increased in asthma with incompletely reversible airflow obstruction. Exp Lung Res 2009;35:781–94. 19916861 10.3109/01902140902906412

[61] Russell CD, Valanciute A, Gachanja NN, Stephen J, Penrice-Randal R, Armstrong SD, Tissue proteomic analysis identifies mechanisms and stages of immunopathology in fatal COVID-19. Am J Respir Cell Mol Biol 2022;66:196–205. 34710339 10.1165/rcmb.2021-0358OC PMC8845132

[62] Lai Y-J, Liu S-H, Manachevakul S, Lee T-A, Kuo C-T, Bello D. Biomarkers in long COVID-19: a systematic review. Front Med 2023;10:1085988. 10.3389/fmed.2023.1085988 PMC9895110 36744129

[63] Pothoven KL, Norton JE, Suh LA, Carter RG, Harris KE, Biyasheva A, Neutrophils are a major source of the epithelial barrier disrupting cytokine oncostatin M in patients with mucosal airways disease. J Allergy Clin Immunol 2017;139:1966–78. 27993536 10.1016/j.jaci.2016.10.039 PMC5529124

[64] Santos AF, Alpan O, Hoffmann H-J. Basophil activation test: mechanisms and considerations for use in clinical trials and clinical practice. Allergy 2021;76:2420–32. 33475181 10.1111/all.14747

[65] Van Panhuys N, Prout M, Forbes E, Min B, Paul WE, Le Gros G. Basophils are the major producers of IL-4 during primary helminth infection. J Immunol 2011;186:2719–28. 21270410 10.4049/jimmunol.1000940 PMC3488853

[66] Wakahara K, Van VQ, Baba N, Bgin P, Rubio M, Delespesse G, Basophils are recruited to inflamed lungs and exacerbate memory Th2 responses in mice and humans. Allergy 2013;68:180–9. 23205591 10.1111/all.12072

[67] Caterina MJ, Schumacher MA, Tominaga M, Rosen TA, Levine JD, Julius D. The capsaicin receptor: a heat-activated ion channel in the pain pathway. Nature 1997; 389:816–24. 9349813 10.1038/39807

[68] Caterina MJ, Rosen TA, Tominaga M, Brake AJ, Julius D. A capsaicin-receptor homologue with a high threshold for noxious heat. Nature 1999;398:436–41. 10201375 10.1038/18906

[69] Szallasi A, Cortright DN, Blum CA, Eid SR. The vanilloid receptor TRPV1: 10 years from channel cloning to antagonist proof-of-concept. Nat Rev Drug Discov 2007;6:357–72. 17464295 10.1038/nrd2280

[70] Mathur S, Wang J-C, Seehus CR, Poirier F, Crosson T, Hsieh Y-C, Nociceptor neurons promote IgE class switch in B cells. JCI Insight 2021;6:e148510. 34727095 10.1172/jci.insight.148510 PMC8783686

[71] Crosson T, Wang J-C, Doyle B, Merrison H, Balood M, Parrin A, FcεR1-expressing nociceptors trigger allergic airway inflammation. J Allergy Clin Immunol 2021;147:2330–42. 33453289 10.1016/j.jaci.2020.12.644 PMC9004488

[72] Talbot S, Abdulnour R-EE, Burkett PR, Lee S, Cronin SJ, Pascal MA, Silencing nociceptor neurons reduces allergic airway inflammation. Neuron 2015;87:341–54. 26119026 10.1016/j.neuron.2015.06.007 PMC4506220

[73] Talbot S, Doyle B, Huang J, Wang J-C, Ahmadi M, Roberson DP, Vagal sensory neurons drive mucous cell metaplasia. J Allergy Clin Immunol 2020;145:1693–6.e4. 31954778 10.1016/j.jaci.2020.01.003 PMC8603302

[74] Mwirigi JM, Sankaranarayanan I, Tavares-Ferreira D, Gabriel KA, Palomino S, Li Y, Expansion of OSMR expression and signaling in the human dorsal root ganglion links OSM to neuropathic pain. bioRxiv preprint April 1, 2025. 10.1101/2025.03.26.645611.

