# Supplementary material for: Basophils activate oncostatin M receptor–expressing vagal sensory neurons
Source: J Allergy Clin Immunol. Author manuscript; Available in PMC 2026 Jul 20. (PMC13384724; doi:10.1016/j.jaci.2025.10.027)
Supplement: 1 [file NIHMS2186702-supplement-1.docx]

**Basophils Activate Oncostatin M Receptor-expressing Vagal Sensory Neurons.**

Jo-Chiao Wang, Ph.D. ^1^, Kicheon Park, Ph.D. ^2^, Anais Roger, Ph.D. ^3^, Amin Reza Nikpoor, Ph.D. ^3^, Theo Crosson, Ph.D. ^1^, Hoon James Sunwoo ^3^, Eva Kaufmann, Ph.D. ^3,5^, Moutih Rafei, Ph.D. ^1^, Eric H. Chang, Ph.D. ^2^, Sebastien Talbot Ph.D.^3,4,#^

^1^ Department of Pharmacology and Physiology, Université de Montréal, Quebec, Canada

^2^ Institute for Bioelectronic Medicine, Feinstein Institutes for Medical Research, Northwell Health, Manhasset, New York, USA

^3^ Department of Biomedical and Molecular Sciences, Queen’s University, Ontario, Canada

^4^ Department of Physiology and Pharmacology, Karolinska Institutet, Stockholm, Sweden

^5^ Meakins-Christie Laboratories, RI-MUHC, and Faculty of Dental Medicine and Oral Health Sciences, McGill University, Montreal, Quebec, Canada

^#^ Lead Contact:

Sebastien Talbot, PhD

Phone: 438-521-3466

Email: [sebas.talbot@gmail.com;](mailto:sebas.talbot@gmail.com) [sebastien.talbot@queensu.ca;](mailto:sebastien.talbot@queensu.ca) [sebastien.talbot@ki.se](mailto:sebastien.talbot@ki.se)

ORCID: 0000-0001-9932-7174

ONLINE REPOSITORY

Supplementary Figure 1. *Mrgprd*, *Prkca* and *Prkcq* expression in JNC neurons.

(**A-F**) *In silico* analysis of the GSE192987 scRNAseq data reveals specific gene expression patterns in jugular neurons. *Mrgprd*^+^ jugular neurons do not express *Trpv1* (**A**), and *Mrgpra3*^+^ neurons do not express *Tac1* (**B**). Further analysis highlights the transcript expression patterns of *Prkaca* (**C**), *Prkca* (**D**), and *Prkcq* (**E-F**), along with those of *Osmr* (**C-E**) and *Mrgprd* (**F**).

*(****A-F****) Data are presented as pseudocolors, representing cells with non-zero counts per ten thousand. The UMAP plots were generated using data from the database GSE192987. Experimental details were outlined in Zhao et al.*^26^

ul. Fig. 1

Supplementary Figure 2. Mouse basophils and human neutrophils express OSM.

(**A-B**) *In silico* analysis of OSM expression in mouse (Immgen; **A**) and human (CELLxGENE; **B**) immune cells reveal its expression in mouse basophils (**A**) and human neutrophils (**B**).

***(A****) Data are presented as normalized gene expression using the median of ratios method. The heatmap plot were generated using data from the database detailed in Gal-Oz ST et al., ^73^*

*(****B****) Data are presented as cluster average of gene expression normalized by counts per ten thousand + 1 of genes having a non-zero value. The heatmap plot were generated using data from the database detailed in Biology CS-C et al., ^74^.*

Su

ppl. Fig. 2

**Supplementary Figure 3. Lung basophils release IL-4 upon FcεRI engagement.**

(**A**) Lung cells (10^6^ cells/well), harvested from naïve C57BL/6 mice, were stimulated with MAR-1 (1 µg/mL), Ba13 (1 µg/mL), or a mixture of isotype control antibodies. IL-4 levels in the supernatant were measured by ELISA at 3- and 24-hours post-stimulation.

(**B–G**) 6–10-week-old male and female C57BL/6 mice were sensitized via intraperitoneal injection of an emulsion containing OVA (200 µg/dose) and aluminum hydroxide (1 mg/dose) on days 0 and 7, followed by intranasal OVA challenge (50 µg/dose) with or without fine particulate matter (FPM, 20 µg/dose) (**B, C, F**). Another set of naïve 8-week-old male and female C57BL/6 mice received intranasal house dust mite (HDM, 20 µg/dose) or *Alternaria alternata* (A.alt, 100 µg/dose) daily from day 0 to day 4 and were challenged on days 7 to 9 (**D, E, G**). After sacrifice, bronchoalveolar lavage fluid (BALF) was collected, and eosinophil (**B, D**) or neutrophil (**C, E**) infiltration was assessed by flow cytometry. *Osm* expression in whole lung lysates (**F, G**) was determined by qPCR.

*Data are presented as mean ± SD* ***(A–G)****.*

- ***(A)*** *Data from a single experiment in which lung cells were pooled from three animals (n = 3 technical replicates per group).*
- ***(B, C, F)*** *Pooled data from three independent experiments: n = 7 in the control group, n = 11 in the OVA group, and n = 13 in the OVA+FPM group.*
- ***(D, E)*** *Representative data from two independent experiments with n = 4 per group.*
- ***(G)*** *Representative data from two independent experiments with n = 5 per group.*

*Statistical analyses were performed using one-way ANOVA with Dunnett’s test (****A, F, G****), comparing treatment groups to the isotype control (****A****), control (****F****), or PBS control (****G****), or with Tukey’s test (****B–E****). Significance is indicated by * for p ≤ 0.05, ** for p ≤ 0.01, *** for p ≤ 0.001, or **** for p ≤ 0.0001.*

Sup

pl. Fig. 3

Supplementary Figure 4. OSM prevents capsaicin-induced desensitization in DRG neurons.

(**A-D**) 6-12 weeks old male and female naïve C57Bl6 mice were euthanized, and DRG (**A-B**) or JNC (**C-D**) neurons were harvested and cultured for 24h. The cells were then loaded with Fura-2AM and sequentially stimulated with IL-31 (100 ng/mL; from *60 to* 120 sec), capsaicin (Caps; 100 nM; from 360 to 370, 660 to 670, and 960 to 970 sec), and KCl (100 mM; from 1260 to 1280 *sec*) and calcium flux recorded. (**A and C**) SES (blue) or 100 ng/ml OSM (red) was flown on the cells between the first and the second capsaicin exposure (420-660 sec). (**B and D**) The second and third capsaicin responses were normalized to each individual neuron’s initial response (at 360-370 sec).

(**E-M**) 6-12 weeks old male and female naïve C57BL6 mice were euthanized, JNC neurons harvested and cultured for 24h in the presence of a vehicle or OSM (100 ng/mL). The cells were harvested, and transcript expressions of *Htr1f* (**F**), *Hrh1* (**G**), *Cysltr2* (**H**), *S1pr1* (**I**), *Sst* (**J**), *Nppb* (**K**), *Fstl1* (**L**), and *Tac1* (**M**) were assessed by qPCR.

*Data are presented as means ± 95% CI of the maximum Fura-2AM (F/F₀) fluorescence recorded every 15 seconds (****A, C****), as means ± SD (****B, D, F–M****), or as a schematic (****E****).*

*For each experiment, jugular–nodose complexes were harvested from 10 mice and pooled into 10 dishes, while dorsal root ganglia from each animal were cultured in 4 dishes. Dishes were randomly assigned to either the SES or OSM-treated group, and one field of view was collected per dish. Representative data from 2 independent experiments are shown for* ***A-D****. In total, n = 33 capsaicin-responsive SES-treated and n = 15 OSM-treated DRG neurons were analyzed (****A, B****), and n = 49 capsaicin-responsive SES-treated and n = 28 OSM-treated JNC neurons were analyzed (****C, D****). For* ***F-M****, n = 3–9 technical repeats from 1–3 experiments.*

*Statistical analyses were performed using two-way ANOVA with Šidák’s test (****B, D****) or an unpaired t-test (****F–M****) and are indicated as ns (not significant), ** for p ≤ 0.01, or **** for p ≤ 0.0001.*

**SUPPLEMENTARY MATERIALS**

**Buffers, Cell culture media, and supplements**

Supplementary Table 1. Digestion buffer for ganglia dissociation

| **Items** | **Suppliers** | **Catalog** | **Final Conc. in supplemented FBS-free DMEM** |
| --- | --- | --- | --- |
| Collagenase, Type 4 | Worthington | LS004189 | 1 mg/ml (325 U/ml) |
| Dispase II | Sigma-Aldrich | 4942078001 | 2 mg/ml (1.8 U/ml) |
| DNase I | Sigma-Aldrich | 11284932001 | 250 µg/ml (735.25 U/ml) |

Supplementary Table 2. Supplemented DMEM for enzymatic digestion and lung cell culture

| **Items** | **Suppliers** | **Catalog** | **Final Conc.** |
| --- | --- | --- | --- |
| DMEM, high glucose | Gibco | 11965092 | - |
| Sodium Pyruvate | Gibco | 11360070 | 1 mM |
| GlutaMAX™ Supplement | Gibco | 35050061 | 2 mM |
| Penicillin-Streptomycin Solution | Corning | 30-002-CI | 100 U/mL; 100 µg/mL |
| HEPES | Gibco | 15630080 | 10 mM |
| *FBS, Premium Canadian Origin, Heat Inactivated | Wisent | 90450 | 1:10 |

*Only added during lung cell culture, but not during digestion

Supplementary Table 3. Neuron culture process

| **Items** | **Suppliers** | **Catalog** | **Final Conc. in PBS** |
| --- | --- | --- | --- |
| Bovine Serum Albumin (BSA) | Hyclone | SH30574.02 | 150 mg/ml |
| Laminin | Sigma-Aldrich | L2020 | 50 µg/ml |
| Poly-D-lysine hydrobromide | Sigma-Aldrich | P6407 | 100 µg/ml |
| PBS, pH 7.4 | Gibco | 10-010-049 | - |

Supplementary Table 4. Neuron culture media

| **Items** | **Suppliers** | **Catalog** | **Final Conc.** |
| --- | --- | --- | --- |
| Neurobasal-A medium | Gibco | 10888022 | - |
| Pen/Strep | Corning | 30-002-CI | 100 U/mL; 100 µg/mL |
| GlutaMAX | Gibco | 35050061 | 2 mM |
| HEPES | Gibco | 15630080 | 10 mM |
| B-27 | Gibco | 17504-044 | 1: 50 |
| Mouse NGF 2.5S Native Protein | Gibco | 13257-019 | 50 ng/ml |
| Recombinant Mouse GDNF Protein | Novus | NBP2-61336 | 2 ng/ml |
| Cytosine-beta-D-arabinofuranose hydrochloride | Thermo Scientific | J6567106 | 10 µM |

Supplementary Table 5. Digestion buffer for lung dissociation

| **Items** | **Suppliers** | **Catalog** | **Final Conc. in supplemented FBS-free DMEM** |
| --- | --- | --- | --- |
| Collagenase, Type 4 | Worthington | LS004189 | 1.6 mg/ml (520 U/ml) |
| DNase I | Sigma-Aldrich | 11284932001 | 100 µg/ml (294.1 U/ml) |

**Reagents**

Supplementary Table 6. Flow Cytometry and Cell Stimulation Antibodies

| **Items** | **clones** | **Suppliers** | **Catalog** | **Final Conc.** | **Experiments** |
| --- | --- | --- | --- | --- | --- |
| Brilliant Violet 421™ anti-mouse CD45 Antibody | 30-F11 | Biolegend | 103134 | 1:400 | Flow cytometry |
| PerCP/Cyanine5.5 anti-mouse CD90.2 (Thy-1.2) Antibody | 53-2.1 | Biolegend | 140322 | 1:400 | Flow cytometry |
| APC/Fire™ 750 anti-mouse/human CD11b Antibody | M1/70 | Biolegend | 101262 | 1:400 | Flow cytometry |
| FITC anti-mouse CD11c Antibody | N418 | Biolegend | 117306 | 1:400 | Flow cytometry |
| PE/Cyanine7 anti-mouse Ly-6C Antibody | HK1.4 | Biolegend | 128018 | 1:400 | Flow cytometry |
| APC anti-mouse Ly-6G Antibody | 1A8 | Biolegend | 127614 | 1:400 | Flow cytometry |
| CD170 (Siglec F) Monoclonal Antibody (1RNM44N), PE | 1RNM44N | eBioscience | 12-1702-82 | 1:400 | Flow cytometry |
| APC anti-mouse CD49b (pan-NK cells) Antibody | DX5 | Biolegend | 108910 | 1:200 | Flow cytometry |
| APC/Fire™ 750 anti-mouse FcεRIα Antibody | MAR-1 | Biolegend | 134340 | 1:100 | Flow cytometry |
| PE/Cyanine7 anti-mouse CD117 (c-Kit) Antibody | 2B8 | Biolegend | 105814 | 1:200 | Flow cytometry |
| FITC anti-mouse CD3*ε* Antibody | 145-2C11 | Biolegend | 100306 | 1:400 | Flow cytometry |
| FITC anti-mouse CD19 Antibody | 1D3/CD19 | Biolegend | 152404 | 1:400 | Flow cytometry |
| FITC anti-mouse NK-1.1 Antibody | PK136 | Biolegend | 108706 | 1:400 | Flow cytometry |
| Biotin anti-mouse FcεRIα Antibody | MAR-1 | Biolegend | 134304 | 1 µg/ml | *in vitro* stimulation |
| Ultra-LEAF™ Purified anti-mouse CD200R3 Antibody | Ba13 | Biolegend | 142215 | 1 µg/ml | *in vitro* stimulation |
| Biotin Armenian Hamster IgG Isotype Ctrl Antibody | HTK888 | Biolegend | 400903 | 1 µg/ml | *in vitro* stimulation |
| Ultra-LEAF™ Purified Rat IgG2a, κ Isotype Ctrl Antibody | RTK2758 | Biolegend | 400543 | 1 µg/ml | *in vitro* stimulation |

Cytometer configurations

Supplementary Table 7. BD FACSCanto II

| **Lasers (nm)** | **LP mirrors (nm)** | **BP Filters (nm)** |
| --- | --- | --- |
| 488 | 735  655  556  502 | 780/60  670 (LP)  585/42  530/30  488/10 |
| 633 | 735 | 780/60  660/20 |
| 405 | 502 | 510/50  450/50 |

Supplementary Table 8. Beckman Coulter CytoFLEX

| **Lasers (nm)** | **BP Filters/Mirrors (nm)** |
| --- | --- |
| 405 | 405  450/45  525/40  610/20  660/20  780/60 |
| 488 | 488/8  525/40  690/50 |
| 561 | 561  585/42  610/20  690/50  780/60 |
| 633 | 660/20  712/35  780/60 |

Supplementary Table 9. BD FACSAria III

| **Lasers (nm)** | **LP mirrors (nm)** | **BP Filters (nm)** |
| --- | --- | --- |
| 488 | 735LP  685LP  655LP  600LP  556LP  502LP  530LP | 780/60  695/40  670/14  610/20  575/26  530/30  550/30 |
| 633 | 735LP  690LP | 780/60  710/40  660/20 |
| 405 | 502LP | 530/30  450/40 |

Supplementary Table 10. BD FACSAria Fusion

| **Laser (nm)** | **LP mirrors (nm)** | **BP Filters (nm)** |
| --- | --- | --- |
| 488 | 735  655  610  556  502 | 780/60  695/40  616/23  585/42  530/30 |
| 640 | 755  690 | 780/60  730/60 |
| 405 | 595  505 | 610/20  525/50 |

Supplementary Table 11. Drugs for *in vitro* calcium imaging

| **Items** | **Suppliers** | **Catalog** | **Final Conc.** | **Duration of incubation/injection** |
| --- | --- | --- | --- | --- |
| Fura-2 Leakage Resistant AM | Cayman Chemical | 34993 | 5 µM | 40 minutes |
| xy-17 | Echelon Biosciences | L-9118 | 10 µM | 25 seconds |
| RS 56812 hydrochloride | Alomone | R-130 | 1 µM | 15 seconds |
| β-Alanine | Sigma-Aldrich | 146064 | 1 mM | 20 seconds |
| Histamine | Cayman Chemical | 33828 | 50 µM | 60 seconds |
| Chloroquine diphosphate salt | Sigma-Aldrich | C6628 | 1 mM | 30 seconds |
| (E)-Capsaicin | Tocris | 0462 | 0.1-1 µM | 10 seconds |
| Potassium Chloride | VWR | 0395 | 40-100 mM | 20 seconds |
| Recombinant Murine IL-31 | Peprotech | 210-31-100UG | 100 ng/ml | 60 seconds |
| Recombinant Mouse Oncostatin M (OSM) Protein | R&D system | 495-MO | 100 ng/ml | 4 minutes |

Supplementary Table 12. Primers for qPCR

| **Genes** | **Forward primer** | **Reverse primer** |
| --- | --- | --- |
| *Osm* | CTAAGAACACTGCTCAGTTTGACC | TGATTCTGTGTTCCCCGTGAG |
| *Trpv1* | GGCCGAGTTTCAGGGAGAAA | TATCTCGAGTGCTTGCGTCC |
| *Mrgpra3* | GTATCCTTCCTTCTACACAAGCC | CTGCACTGGTGTTGCTTTCT |
| *Il6st* | CTGAGGGACCGGTGGTGTG | TCCTTCTATCGGGTCTTCCTTCC |
| *Il31ra* | TGGACCATCGAGCAAGATAAACT | ACCCTGGTCTCAGGACCTTT |
| *Il11ra1* | GTTGCAATACCGACCAGCAC | CCAGCCACAGCATCTGTTAT |
| *Cntfr* | TGTTTCCACCGTGACTCCTG | AGCTGCAGTAGAAGCCCTTG |
| *Htr1f* | GTTGTCGCCAGAGAACGACC | CTCAACTCAGCTTCCCCAGAG |
| *Hrh1* | ACTCAGCCACGAGTGAAACC | GTGATGGCTCCCTCCCTCG |
| *Cysltr2* | CTGCATTCTATGGGGCGAGA | CAGGTACATGTAAAAGACTCTGAAC |
| *S1pr1* | GCATTAACCCCTCCCAGTCC | AGAAACAGCAGCCTCGCTC |
| *Sst* | GACCCCAGACTCCGTCAGTT | GTACTTGGCCAGTTCCTGTTC |
| *Tac1* | GTGACCAGATCAAGGAGGCA | ATGTCCAGCATCCCGCTTG |
| *Fstl1* | CACGATGTGGAAACGATGGC | TTCTAGGTTCCTCCTCGCCG |
| *Nppb* | ATCTCAAGCTGCTTTGGGCA | ACTTCAGTGCGTTACAGCC |

Supplementary Table 13. ELISA kits

| **Items** | **Suppliers** | **Catalog** |
| --- | --- | --- |
| ELISA MAX™ Standard Set Mouse IL-4 | Biolegend | 431101 |
| ELISA MAX™ Deluxe Set Mouse LIF | Biolegend | 445104 |
| Mouse Oncostatin M (OSM) DuoSet ELISA | R&D system | DY495-05 |
